# Conservation of Forces and Total Work at the Interface Using the Internodes Method

**DOI:** 10.1007/s10013-022-00560-9

**Published:** 2022-05-10

**Authors:** Simone Deparis, Paola Gervasio

**Affiliations:** 1 Institute of Mathematics, École Polytechnique Fédérale de Lausanne (EPFL), Lausanne, Switzerland; 2 DICATAM, Università degli Studi di Brescia, Brescia, Italy

**Keywords:** Domain decomposition, Non-conforming coupling, Conservation properties, Finite element method, *hp*-finite element method, Spectral element method, 65N55, 65N30, 65N12, 65D05

## Abstract

The Internodes method is a general purpose method to deal with non-conforming discretizations of partial differential equations on 2D and 3D regions partitioned into disjoint subdomains. In this paper we are interested in measuring how much the Internodes method is conservative across the interface. If *hp*-fem discretizations are employed, we prove that both the total force and total work generated by the numerical solution at the interface of the decomposition vanish in an optimal way when the mesh size tends to zero, i.e., like $\mathcal {O}(h^{p})$, where *p* is the local polynomial degree and *h* the mesh-size. This is the same as the error decay in the *H*^1^-broken norm. We observe that the conservation properties of a method are intrinsic to the method itself because they depend on the way the interface conditions are enforced rather then on the problem we are called to approximate. For this reason, in this paper, we focus on second-order elliptic PDEs, although we use the terminology (of forces and works) proper of linear elasticity. Two and three dimensional numerical experiments corroborate the theoretical findings, also by comparing Internodes with Mortar and WACA methods.

## Introduction

We are interested in the approximation of partial differential equations by non-conforming domain decomposition methods, more specifically in the conservation properties at the interface between non-overlapping subdomains. The non-conformity at the interface can be induced by different independent discretizations inside the subdomains, as well as by non-watertight interfaces.

Is the traction and the work at exact equilibrium across the interface? If not, how well it is, and what is the dependence on the mesh refinement? And maybe even more importantly, which quantities may-be expected to be conserved at the interface?

Among the non-conforming coupling methods, the most widely used and studied is the Mortar method (far from be exhaustive see, e.g., [3–5, 7, 9, 29]), which is optimal in terms of convergence rates and is based on a single *L*^2^ projection operator; as we will see throughout the paper, the Mortar method also conserves traction forces and works along the interface; see also [14] for a discussion in the case of fluid-structure interation problems.

Differently from the Mortar method, the Internodes (INTERpolation for NOnconforming DEcompositionS) method is based on two intergrid interpolation operators, one for transferring the Dirichlet trace across the interface, the others for transferring the Neumann trace, i.e., the fluxes or the stresses. The Internodes method has been proposed in [11] in the context of second order elliptic differential problems, and it has been applied successfully to fluid-structure interaction problems [13], Navier–Stokes equations [11], Stokes–Darcy coupling [18]. Its analysis for 2D and 3D second-order elliptic equations has been carried out in [16] and its generalization to decompositions with more than two subdomains is presented in [16, 19]. The Internodes method has also been applied in the context of Isogeometric Analysis [15] to deal with non-conforming multi-patch geometries. For what concerns the theoretical analysis, it has been proved in [16] that, when the mesh sizes *h*_1_ and *h*_2_ of the two subdomains vanish with the same rate and the local polynomial degree *p* (the same in both subdomain) grows up, the Internodes method features the same convergence order of the Mortar method in the *H*^1^-broken norm error. More precisely, it holds that the global *H*^1^-broken norm error behaves like $\mathcal {O}(h^{p})$, with $h=\max \limits \{h_{1},h_{2}\}$. When this happens, it is said that the convergence order of the multidomain method is optimal. The spectral properties of the algebraic form of the Internodes method have been investigated in [11, Sect. 6] and compared with those of the Mortar method. Numerical results show that the extreme moduli of the eigenvalues of the global Internodes matrix, associated with the discretization of the Laplace operator, behave like the analogous quantities related to the Mortar method.

We point out that the Internodes method works with two independent interpolation matrices, and this choice guarantees to keep the optimal convergence order of the local discretizations inside the two subdomains. On the contrary, the *consistent interpolating* approach discussed in [14], that coincides with the *pointwise* interpolation method analysed in [6], features a degradation of the solution of the coupled problem with respect to the mesh-size and to the polynomial degree, although it is conservative across the interface.

Alternatives are Nietsche [1] or discontinuous Galerkin approaches, in which jumps between domains are evaluated, and that needs interpolation or projection operators between subdomains for the computation of integrals of quantities of both domains simultaneously. The Internodes method needs an interpolation operator between the subdomains but there is no need of crossed integrals.

In this paper we show that the Internodes method conserves both traction forces and works across the interface, in the sense that the total force and the total work at the interface vanish at least like the global broken-norm error when the discretizations in both subdomains are refined.

The conservation properties of some coupling methods have been reviewed in [10]. In the present paper, we try to give a clear characterisation of what *conservative* method means by defining the quantities that a coupling method should conserve (asymptotically or exactly); then, on one hand we provide a mathematical proof of the conservation properties of the Internodes method, on the other hand we yield numerical experiments showing the conservation properties of the Mortar method, the Internodes method, and the weighted average continuity approach (WACA) proposed in [10].

In [22] the authors addressed the problem of conserving the energy across an interface in the framework of non-conforming interfaces between a finite volume and FEM; they consider that the energy is conserved if the work is equal on both sides of the interface.

We observe that the conservation properties of a method are intrinsic to the method itself because they depend on the way the interface conditions are enforced rather than on the problem we are called to approximate. For this reason we focus on second order elliptic partial differential equations (PDEs) and after decompositon of the computational domain into two subregions, we analyse the conservation properties across the common interface; this interface can be either conforming or non-conforming depending on the local space discretisations and on the meshes. In the continuous settings, the total force and the total work across the interface are zero, both when computed as integrals (in strong form) on the interface and as sum of residuals (weak form); indeed they coincide thanks to the Green formula. When using classical conforming methods based on the Galerkin projection [26, 28], the sums of residuals are null while the strong forms asymptotically vanish when the mesh-size tends to zero.

In case of non-conforming methods, one has to first establish how to compute the total force and work, since the interface on the two sides may be different (geometric non-conforming, see, e.g., the characterization given in [11]). In this case the integral forms and the residual ones do not coincide anymore. The Mortar method guarantees that the residual forms of total work and total force are zero, however the strong ones are “only” optimally convergent, but not identically null. By optimal convergence we mean here the *H*^1^-broken-norm convergence order that behaves like the worst *best approximation error* inside the two subdomains and that is generally used to measure the approximation properties of non-conforming methods.

In Section 4 we prove that Internodes provides optimal convergence for both versions (strong and residual-based) of the total force and work. We have performed two and three dimensional experiments using geometrical conforming and non-conforming decompositions and different polynomial degrees, by using finite element or spectral element methods. We have compared Mortar, Internodes, and WACA methods. Mortar and Internodes provide optimal order convergence (when not exact, as explained above), whilst WACA allows for exact residual versions of the total quantities, but at most for first order convergence for the strong forms, even for high order approximations.

The paper is structured as follows. In Section 2, we briefly recall the mathematical setting of elliptic PDE’s in a two subdomains framework in either 2D or 3D domains and, by inheriting the terminology from linear elasticity, we define the quantitites that we want to analyse in order to evaluate the conservation properties of a multidomain method, i.e., the total force and the total work. In Section 3 we recall the Internodes method and its approximation properties. Section 4 is devoted to the analysis of the conservation properties of the Internodes method and in Section 5 we provide numerical evidence to the conservation and approximation properties of the Internodes and Mortar methods by reporting numerical results in both 2D and 3D domains.

## Problem Formulation

**Fig. 1:**
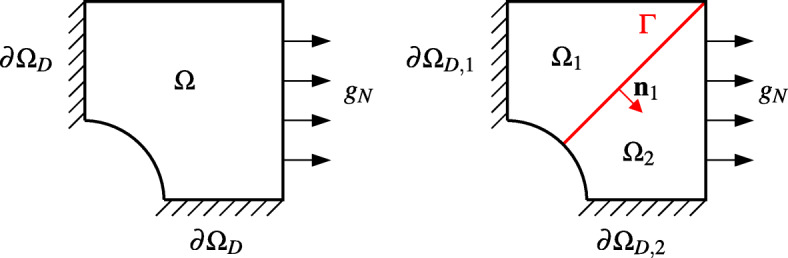
The computational domain (on the left) and its decomposition (on the right)

Let us consider a self-adjoint second order elliptic problem, for which we seek the function $u:{{{\varOmega }}}\to \mathbb {R}$ solution of

1 $$ \begin{array}{@{}rcl@{}} \left\{\begin{array}{ll} Lu=-\nabla\cdot(\nu\nabla u)+\gamma u=f & \text{ in }{{{\varOmega}}}\subset{\mathbb{R}}^{d},\\ u=0 & \text{ on }\partial{{{\varOmega}}}_{D},\\ \nu \frac{\partial u}{\partial \textbf{n}}=g_{N}& \text{ on }\partial{{{\varOmega}}}_{N}, \end{array}\right. \end{array} $$

where ${{{\varOmega }}}\subset {\mathbb {R}}^{d}$, with *d* = 2,3, is an open domain with Lipschitz boundary *∂Ω*, *ν* > 0, *γ* ≥ 0, and *f* are given functions defined in *Ω*, while *g*_*N*_ is a given function defined on $\partial {{{\varOmega }}}_{N}\subseteq \partial {{{\varOmega }}}$ (*∂Ω*_*N*_ and $\partial {{{\varOmega }}}_{D}\subseteq \partial {{{\varOmega }}}$ are such that $\overline {\partial {{{\varOmega }}}} = \overline {\partial {{{\varOmega }}}_{D}\cup \partial {{{\varOmega }}}_{N}}$ and *∂Ω*_*D*_ ∩ *∂Ω*_*N*_ = *∅*), and finally **n** is the outward unit normal vector to *∂Ω*. (See Fig. 1, left.) Non-homogeneous Dirichlet conditions can be dealt with by standard arguments (see, e.g., [25]).

We split *Ω* into two disjoint subdomains *Ω*_1_ and *Ω*_2_ such that $\overline {{{{\varOmega }}}_{1}\cup {{{\varOmega }}}_{2}}=\overline {{{{\varOmega }}}}$ and we denote the common interface by *Γ* = *∂Ω*_1_ ∩ *∂Ω*_2_ (see Fig. 1, right).

The multidomain formulation of (1) reads (see, e.g., [26, 28]): for *k* = 1,2 look for the functions *u*_*k*_ defined on *Ω*_*k*_ such that

2 $$ \begin{array}{@{}rcl@{}} \left\{\begin{array}{ll} -\nabla\cdot(\nu\nabla u_{k})+\gamma u_{k}=f & \text{ in }{{{\varOmega}}}_{k}, \quad k=1,2,\\ u_{1}=u_{2}, & \text{ on }{{{\varGamma}}},\\ \nu\frac{\partial u_{1}}{\partial \textbf{n}_{1}} + \nu\frac{\partial u_{2}}{\partial \textbf{n}_{2}} =0 & \text{ on }{{{\varGamma}}},\\ +\text{ boundary conditions inhereted from (1)} & \text{ on }\partial{{{\varOmega}}}_{k}\cap \partial{{{\varOmega}}}, \end{array}\right. \end{array} $$

where **n**_*k*_ is the outward unit normal vector to *∂Ω*_*k*_, while *∂Ω*_*k*, *D*_ = *∂Ω*_*D*_ ∩ *∂Ω*_*k*_ and *∂Ω*_*k*, *N*_ = *∂Ω*_*N*_ ∩ *∂Ω*_*k*_ are the Dirichlet and Neumann boundaries, respectively, restricted to the domain *Ω*_*k*_. Condition (2)_2_ enforces the continuity of the solution across the interface *Γ*, while (2)_3_ enforces the balance of the normal derivatives on *Γ*.

To write the weak form of (2) we define the spaces

$$ \begin{array}{@{}rcl@{}} V_{k} &=& \{v\in H^{1}({{{\varOmega}}}_{k}):~v|_{\partial{{{\varOmega}}}_{k,D}}=0\}, \text{ for }k=1,2,\\ {V_{k}^{0}} &=& \{v_{k}\in V_{k}:~ v_{k}|_{{{{\varGamma}}}}=0\}, \text{ for }k=1,2,\\ {{{\varLambda}}} &=& \{\lambda\in H^{1/2}({{{\varGamma}}}):~ \exists v\in H^{1}_{0,\partial{{{\varOmega}}}_{D}}({{{\varOmega}}}): v|_{{{{\varGamma}}}}=\lambda\}, \end{array} $$

where $H^{1}_{0,\partial {{{\varOmega }}}_{D}}({{{\varOmega }}})=\{v\in H^{1}({{{\varOmega }}}):~v|_{\partial {{{\varOmega }}}_{D}}=0\}$. *Λ* is the space of traces of the functions of *V* on the interface.

Provided that *f* ∈ *L*^2^(*Ω*), $g_{N}\in L^{2}(\partial {{{\varOmega }}}_{N})$, and $\nu , \gamma \in L^{\infty }({{{\varOmega }}})$, the weak form of (2) reads: for *k* = 1,2 find *u*_*k*_ ∈ *V*_*k*_ such that

3 $$  \left\{\begin{array}{ll} \displaystyle{\int}_{{{{\varOmega}}}_{k}} \nu \nabla u_{k}\cdot \nabla v_{k} + \gamma u_{k} v_{k} = {\int}_{{{{\varOmega}}}_{k}} f v_{k}+ {\int}_{\partial{{{\varOmega}}}_{k,N}}\!\!\! g_{N} v_{k} &\quad \forall v_{k}\in {V_{k}^{0}}, \quad k=1,2,\\ u_{1}=u_{2} &\quad \text{ on }{{{\varGamma}}},\\ \displaystyle{\int}_{{{{\varGamma}}}} \nu \frac{\partial u_{1}}{\partial\textbf{n}_{1}}\mu+ {\int}_{{{{\varGamma}}}} \nu \frac{\partial u_{2}}{\partial\textbf{n}_{2}}\mu=0 &\quad \forall \mu \in {{{\varLambda}}}, \end{array}\right.  $$

where the integrals in the last equation must be interpreted as dualities between the trace space *Λ* and its dual space ${{{\varLambda }}}^{\prime }$.

The classical abstract form of (3) reads: for *k* = 1,2, find *u*_*k*_ ∈ *V*_*k*_ such that

4 $$ \begin{array}{@{}rcl@{}} \left\{\begin{array}{ll} a_{k}(u_{k},v_{k})={\mathcal{F}}_{k}(v_{k}) & \forall v_{k}\in {V_{k}^{0}}, \quad k=1,2,\\ \displaystyle u_{1}=u_{2} & \text{ on }{{{\varGamma}}},\\ \displaystyle\sum\limits_{k=1,2} a_{k}(u_{k},{\mathcal{R}}_{k} \mu)=\sum\limits_{k=1,2} {\mathcal{F}}_{k}({\mathcal{R}}_{k} \mu) & \forall \mu \in {{{\varLambda}}}, \end{array}\right. \end{array} $$

where $a_{k}(u_{k},v_{k})={\int \limits }_{{{{\varOmega }}}_{k}} \nu \nabla u_{k}\cdot \nabla v_{k}+\gamma u_{k} v_{k}$, ${\mathcal {F}}_{k}(v_{k})={\int \limits }_{{{{\varOmega }}}_{k}}f v_{k}+ {\int \limits }_{\partial {{{\varOmega }}}_{k,N}}g_{N} v_{k}$ and ${\mathcal {R}}_{k}:{{{\varLambda }}}\to V_{k}$ denotes any linear and continuous lifting operator from the interface to the domain *Ω*_*k*_.

It is well known (see, e.g. [26]) that problem (4) admits a unique solution and that *u*_1_ and *u*_2_ are the restrictions to *Ω*_1_ and *Ω*_2_, respectively, of the weak solution of (1).

The conditions (3)_3_ (that should be interpreted as duality when the normal derivatives are not sufficiently regular) and (4)_3_ are the weak counterpart of (2)_3_ and, by choosing the test function *μ* in a suitable way, they express two fundamental balance principles at the interface, that we are going to describe below. We make the following assumptions that we comment at the end of the present section, see Remark 1.

### **Assumption 1**

Let us assume that Neumann boundary conditions are imposed on that part of *∂Ω* that matches the interface *Γ* (i.e., we assume that *∂Γ* ∩ *∂Ω*_*D*_ = *∅*).

Let Assumption 1 be satisfied. By adopting the terminology of linear elasticity (in which the Laplace operator is replaced by the divergence of the Cauchy stress tensor), if we choose *μ* ≡ 1 in (3)_3_ we obtain a sort of “balance of forces” at the interface, that reads

5 $$  TF = {\int}_{{{{\varGamma}}}} \nu\frac{\partial u_{1}}{\partial\textbf{n}_{1}}+ {\int}_{{{{\varGamma}}}} \nu\frac{\partial u_{2}}{\partial\textbf{n}_{2}}=0,  $$

where *TF* stands for *Total Force*. This terminology is inherited from linear elasticity, where the normal derivative of the solution on the boundary is the normal component of the stress tensor and expresses a traction or a normal force to the boundary.

If instead we choose *μ* = *u*_1__|*Γ*_ = *u*_2__|*Γ*_ (in virtue of (3)_2_), we obtain a sort of “null total work” on *Γ*, i.e,

6 $$  TW={\int}_{{{{\varGamma}}}}\nu \frac{\partial u_{1}}{\partial\textbf{n}_{1}}u_{1}+ {\int}_{{{{\varGamma}}}} \nu\frac{\partial u_{2}}{\partial\textbf{n}_{2}}u_{2}=0,  $$

here *TW* stands for *Total Work*. Also here we refer to linear elasticity, where the product $\nu \frac {\partial u}{\partial \textbf {n}}u$ is replaced by the product between the normal stress and the displacement, thus giving a work.

Finally, we notice that by setting *λ* = *u*_1__|*Γ*_ = *u*_2__|*Γ*_ in (6) and by applying the first Green formula, the identities (5) and (6) can be equivalently written as

7 $$ \begin{array}{@{}rcl@{}} TF_{a} &=& \sum\limits_{k=1,2}\left[a_{k}(u_{k}, \mathcal{R}_{k} 1))-\mathcal{F}_{k}(\mathcal{R}_{k} 1))\right]=0,  \end{array} $$

8 $$ \begin{array}{@{}rcl@{}} TW_{a} &=& \sum\limits_{k=1,2}\left[a_{k}(u_{k}, \mathcal{R}_{k}\lambda))-\mathcal{F}_{k}(\mathcal{R}_{k}\lambda)\right]=0.  \end{array} $$

In fact, they are *sum of residuals* and are particular instances of the equation (4)_3_ with *μ* = 1 and *μ* = *u*_|*Γ*_, respectively. Formulas (5) and (6) are what we call *strong forms* of the total force and total work, respectively, while (7) and (8) are the corresponding *residual* or *weak forms*.

The balance of forces (5), or (7), and the null total work (6), or (8), express the conservation for the multidomain problem (3).

When the multidomain problem (3) is approximated (by either a conforming or a non-conforming method) it is desiderable that the discrete solution satisfies the discrete counterpart of (5)–(8) up to a term that vanishes with a certain order *q* with respect to the discretization parameters, e.g. the mesh size of the discretization. If that happens, we say that the *multidomain approach is conservative of order* *q* with respect to the mesh size. We refer to Section 4, Definition 1 for a formal definition of this concept.

In this paper, we show that when we combine Internodes with Finite Elements or Spectral Elements, we obtain a conservative multidomain discrete approach and that the order of conservation equals the order of the broken-norm error (with respect to the mesh size).

We notice that conservation of a method is intrinsic to the method itself because conservation depends on the way the interface conditions are enforced rather then on the problem we are called to approximate. Thus, the analysis provided here can be extended to other PDEs for which the conservation at the interface is meaningful.

### *Remark 1*

Assumption 1 is made for two reasons both connected with the theoretical analysis. First of all we notice that, if we set Dirichlet conditions on the part of *∂Ω* that matches the interface *Γ*, then *μ* should belong to ${{{\varLambda }}}=H^{1/2}_{00}({{{\varLambda }}})$ and we could not choose *μ* ≡ 1 in (3)_3_. This would imply that the conservation of forces could not be brought back to the interface condition (3)_3_. The second reason is related to the convergence analysis of the Internodes method that, at the moment, is available provided that Assumption 1 is satisfied.

However, we can infer from the numerical results of Section 5 that the conservation properties of the Internodes method are kept even when Assumption 1 is not satisfied.

## Internodes for *hp*-fem Discretization

We sketch here the idea of Internodes and we refer to [11, 16, 19] for an exahustive presentation of the method for what concerns theoretical properties, algebraic formulation, and algorithmic aspects.

We consider two a-priori *independent families of triangulations* $\mathcal {T}_{1,h}$ in *Ω*_1_ and $\mathcal {T}_{2,h}$ in *Ω*_2_, respectively, characterized by different mesh-sizes *h*_1_ and *h*_2_. This means that the meshes in *Ω*_1_ and in *Ω*_2_ can be non-conforming on *Γ*. The elements can be either simplices (triangles if *d* = 2 or tetrahedra if *d* = 3) or quads (i.e. quadrilaterals if *d* = 2 or hexahedra if *d* = 3). Moreover, different polynomial degrees *p*_1_ and *p*_2_ can be used to define the finite element spaces on $\mathcal {T}_{1,h}$ and $\mathcal {T}_{2,h}$. Inside each subdomain *Ω*_*k*_ we assume that the triangulations $\mathcal {T}_{k,h}$ are regular and quasi-uniform [25, Chapter 3]. Moreover, we assume that they are affine when simplices are considered. We denote by *Γ*_1_ and *Γ*_2_ the internal boundaries of *Ω*_1_ and *Ω*_2_, respectively, induced by the triangulations $\mathcal {T}_{1,h}$ and $\mathcal {T}_{2,h}$. If *Γ* is flat, then *Γ*_1_ = *Γ*_2_ = *Γ*, otherwise *Γ*_1_ and *Γ*_2_ can be different. The finite element approximation spaces (for *k* = 1,2) are

$$ \begin{array}{@{}rcl@{}} X_{k,h} &=& \left\{v \in C^{0}(\overline{{{{\varOmega}}}}_{k})~~~:~ v_{|T_{m}} \in \mathcal{Q}_{p_{k}},~ \forall T_{m} \in {\mathcal{T}}_{k,h}\right\},\\ V_{k,h} &=& X_{k,h}\cap V_{k}, \qquad V_{k,h}^{0}=X_{k,h}\cap {V_{k}^{0}}, \end{array} $$

where $\mathcal {Q}_{p_{k}}={\mathbb {P}}_{p_{k}}$ if the *T*_*m*_ are simplices and $\mathcal {Q}_{p_{k}}={\mathbb {Q}}_{p_{k}} \circ \textbf {F}^{-1}_{T_{m}}$ if the *T*_*m*_ are quads; the corresponding spaces of traces on the interfaces are

$$ Y_{k,h} = \{\lambda = v|_{{{{\varGamma}}}_{k}},~v\in X_{k,h}\}\quad \text{ and }\quad {{{\varLambda}}}_{k,h}=\{\lambda=v|_{{{{\varGamma}}}_{k}},~v\in V_{k,h}\}. $$

In order to exchange information between the two independent grids on the interface *Γ*, we introduce two independent *interpolation operators*:

9 $$  {{{\varPi}}}_{12}:Y_{2,h}\to Y_{1,h}\quad \text{ and } \quad {{{\varPi}}}_{21}:Y_{1,h}\to Y_{2,h}  $$

that are going to interpolate the derivatives and traces from one interface to the other one. These arguments apply both in two and three dimensions.

When *Γ*_1_ and *Γ*_2_ coincide, then *π*_12_ and *π*_21_ are the classical Lagrange interpolation operators (see [11, 16]), otherwise *π*_12_ and *π*_21_ can be defined as the Rescaled Localized Radial Basis Function (RL-RBF) interpolation operators introduced in formula (3.1) of [12] (see also [19, Sect. 2.2.2]).

Obviously, in the conforming case for which *Γ*_1_ = *Γ*_2_, *h*_1_ = *h*_2_ and *p*_1_ = *p*_2_, the interpolation operators *π*_12_ and *π*_21_ are the identity operator.

The Internodes method applied to (1) reads: find *u*_1,*h*_ ∈ *V*_1,*h*_ and *u*_2,*h*_ ∈ *V*_2,*h*_, such that

10 $$  \left\{\begin{array}{ll} a_{k}(u_{k,h},v_{k,h})={\mathcal{F}}_{k}(v_{k,h})&\quad\forall v_{k,h}\in V^{0}_{{k,h}},\quad k=1,2 \\ u_{2,h}={{{\varPi}}}_{21}u_{1,h} &\quad \text{ on }{{{\varGamma}}}_{2},\\ r_{1,h}+{{{\varPi}}}_{12} r_{2,h}=0 &\quad \text{ on }{{{\varGamma}}}_{1}, \end{array}\right.  $$

where for *k* = 1,2, $r_{k,h}\in Y_{k,h}^{\prime }(=Y_{k,h})$ is the so-called *residual at the interfaceΓ*_*k*_, and it is defined starting from the solutions *u*_*k*, *h*_ as follows:

- set the real values
  11 $$  r_{k,i} = a_{k}\left( u_{k,h},\overline{\mathcal{R}}_{k} \mu_{i}^{(k)}\right) - \mathcal{F}_{k}\left( \overline{\mathcal{R}}_{k} \mu_{i}^{(k)}\right), \qquad i=1,{\ldots} n_{k},  $$
  where $\{\mu _{i}^{(k)}\}_{i=1}^{n_{k}}$ is the Lagrange basis in *Y*_*k*, *h*_ and $\overline {\mathcal {R}}_{k}\mu _{i}^{(k)}\in X_{k,h}$ is the *finite element extension* to *Ω*_*k*_ of $\mu _{i}^{(k)}\in Y_{k,h}$ (that is the function in *X*_*k*, *h*_ that is null at all nodes of $\mathcal {T}_{k,h}$ not belonging to *Γ*_*k*_ and that coincides with $\mu _{i}^{(k)}$ on *Γ*_*k*_),
- define the functions
  12 $$  r_{k,h}= \sum\limits_{i=1}^{n_{k}} r_{k,i} {{{\varPhi}}}_{i}^{(k)},  $$
  where $\{{{{\varPhi }}}_{i}^{(k)}\}_{i=1}^{n_{k}}$ is the canonical *dual basis* of the Lagrange primal basis $\{\mu _{i}^{(k)}\}_{i=1}^{n_{k}}$, i.e., satisfying
  $$ \left\langle {{{\varPhi}}}_{i}^{(k)}, \mu_{j}^{(k)}\right\rangle=\left( {{{\varPhi}}}_{i}^{(k)}, \mu_{j}^{(k)}\right)_{L^{2}({{{\varGamma}}}_{k})}=\delta_{ij}. $$

We remark that the expansion (12) with respect to the dual basis is not suitable to apply the Lagrange interpolation, but in order to interpolate *r*_*k*, *h*_ we have to write it with respect to the primal basis. This is possible because $Y^{\prime }_{k,h}$ and *Y*_*k*, *h*_ are the same algebraic space [8]. The matrix that realizes the change from the primal basis to the dual one is the *interface mass matrix* $M_{{{{\varGamma }}}_{k}}$ whose entries are

13 $$  (M_{{{{\varGamma}}}_{k}})_{ij}=\left( \mu_{j}^{(k)},\mu_{i}^{(k)}\right)_{L^{2}({{{\varGamma}}}_{k})}; \qquad i,j=1,\ldots, n_{k},  $$

conversely, $M_{{{{\varGamma }}}_{k}}^{-1}$ realizes the change from the dual to the primal expansion.

Denoting by $\textbf {r}^{(k)}_{{{{\varGamma }}}}$ the array whose entries are the values *r*_*k*, *i*_, we define the array

$$ \textbf{z}^{(k)}_{{{{\varGamma}}}} = M_{{{{\varGamma}}}_{k}}^{-1} \textbf{r}^{(k)}_{{{{\varGamma}}}} $$

whose entries are denoted by *z*_*k*, *j*_ for *j* = 1,…,*n*_*k*_.

Then the primal expansion of the residual function *r*_*k*, *h*_ reads

$$ r_{k,h}= \sum\limits_{j=1}^{n_{k}} z_{k,j} \mu_{j}^{(k)}, $$

and we are going to apply the interpolation to it.

### *Remark 2*

If Assumption 1 are not satisfied and Dirichlet conditions are imposed on the part of *∂Ω* matching the boundary of the interface *Γ*, then formula (11) must be replaced by

14 $$  r_{k,i} = a_{k}\left( u_{k,h},\overline{\mathcal{R}}_{k} \mu_{i}^{(k)}\right) - \mathcal{F}_{k}\left( \overline{\mathcal{R}}_{k} \mu_{i}^{(k)}\right) - {\int}_{G_{k}} \nu\frac{\partial u_{k,h}}{\partial\textbf{n}_{k}} \overline{\mathcal{R}}_{k}\mu_{i}^{(k)},  $$

where *G*_*k*_ = *∂Ω*_*k*_ ∖ (*Γ*_*k*_ ∪ *∂Ω*_*k*, *N*_).

Formula (14) is justified as follows. When a Lagrange basis function $\mu _{i}^{(k)}$ is associated with a node **x**_*i*_ belonging to the boundary of the interface, its extension $\overline {\mathcal {R}}_{k}\mu _{i}^{(k)}$ is indeed also not null on a part of the external boundary *∂Ω*_*k*_ ∖*Γ*_*k*_. Thus, by integrating by parts $a_{k}(u_{k,h},\overline {\mathcal {R}}_{k} \mu _{i}^{(k)})$ and bearing in mind that *r*_*k*, *h*_ must be an approximation of the normal derivative of *u*_*k*, *h*_ on *Γ*_*k*_, we obtain (14).

### Algebraic Form of Internodes

Using standard notations for finite elements, we denote by *A*^(*k*)^ the stiffness matrix associated with the bilinear form *a*_*k*_ and we decompose it into the 2 × 2 block-wise form

$$ A^{(k)} = \left[\begin{array}{cc} {A}_{II}^{(k)} ~&~ {A}_{I{{{\varGamma}}}}^{(k)}\\ A^{(k)}_{{{{\varGamma}}} I} ~&~ A^{(k)}_{{{{\varGamma}}}{{{\varGamma}}}} \end{array}\right] $$

reflecting the partition of the degrees of freedom (d.o.f.) between internal (*I*) and on the interface (*Γ*). (Notice that the set *I* in fact includes also Neumann d.o.f..) Similarly, we define the array **f**^(*k*)^ associated with the functional $\mathcal {F}_{k}$ and we split it into the “internal” ${\textbf {f}}_{I}^{(k)}$ and “interface” part $\textbf {f}^{(k)}_{{{{\varGamma }}}}$.

Then let ${\textbf {u}}_{I}^{(k)}$ and ${\textbf {u}}_{{{{\varGamma }}}}^{(k)}$ be the arrays of the d.o.f. in *Ω*_*k*_ ∖*Γ*_*k*_ and on *Γ*_*k*_, respectively, while $\textbf {r}^{(k)}_{{{{\varGamma }}}}$ is the array of the interface residual introduced before. Finally, let *P*_12_ and *P*_21_ the matrices associated with the interface interpolation operators *π*_12_ and *π*_21_, respectively, introduced in (9).

The algebraic form of the Internodes method reads: find $\textbf {u}^{(1)}=\left [\begin {array}{c} \textbf {u}^{(1)}_{I}\\ {\textbf {u}}_{{{{\varGamma }}}}^{(2)} \end {array}\right ]$ and $\textbf {u}^{(2)}=\left [\begin {array}{c} \textbf {u}^{(2)}_{I}\\ {\textbf {u}}_{{{{\varGamma }}}}^{(2)} \end {array}\right ]$ solutions of

15 $$  \left\{ \begin{array}{ll} {A}_{II}^{(k)} {\textbf{u}}_{I}^{(k)}+{A}_{I{{{\varGamma}}}}^{(k)} {\textbf{u}}_{{{{\varGamma}}}}^{(k)}={\textbf{f}}_{I}^{(k)} & \text{ for }k=1,2,\\ {\textbf{u}}_{{{{\varGamma}}}}^{(2)}=P_{21}{\textbf{u}}_{{{{\varGamma}}}}^{(1)},\\ {\textbf{r}}_{{{{\varGamma}}}}^{(1)}+M_{{{{\varGamma}}}_{1}}P_{12}M_{{{{\varGamma}}}_{2}}^{-1}{\textbf{r}}_{{{{\varGamma}}}}^{(2)} =\textbf{0}. \end{array}\right.  $$

If the meshes are conforming at the interface, then the interpolation matrices *P*_12_ and *P*_21_ are in fact the identity matrix and $M_{{{{\varGamma }}}_{2}}=M_{{{{\varGamma }}}_{1}}$. It follows that the Internodes method (15) reduces to

16 $$  \left\{\begin{array}{ll} {A}_{II}^{(k)} {\textbf{u}}_{I}^{(k)} + {A}_{I{{{\varGamma}}}}^{(k)} {\textbf{u}}_{{{{\varGamma}}}}^{(k)} = {\textbf{f}}_{I}^{(k)} & \text{ for }k=1,2,\\ {\textbf{u}}_{{{{\varGamma}}}}^{(2)} = {\textbf{u}}_{{{{\varGamma}}}}^{(1)},\\ {\textbf{r}}_{{{{\varGamma}}}}^{(1)} + {\textbf{r}}_{{{{\varGamma}}}}^{(2)} = \textbf{0} \end{array}\right.  $$

that is nothing else the algebraic form of a classical substructuring method on two subdomains (see [26, 28]).

### Accuracy of the Internodes Method

Under the assumptions that problem (1) is well posed (see, e.g., [25]), the convergence analysis of the Internodes method with respect to the mesh sizes *h*_1_ and *h*_2_ is carried out in [16]. More precisely, let

$$ u_{h} = \left\{\begin{array}{ll} u_{1,h} & \quad \text{ in }{{{\varOmega}}}_{1},\\ u_{2,h} & \quad \text{ in }{{{\varOmega}}}_{2} \end{array}\right. $$

denote the Internodes solution and let $u\in H^{1}_{0,\partial {{{\varOmega }}}_{D}}({{{\varOmega }}})$ be the solution of the weak monodomain formulation

$$ {\int}_{{{{\varOmega}}}} (\nu \nabla u\cdot \nabla v +\gamma u v) = {\int}_{{{{\varOmega}}}} f v+{\int}_{\partial{{{\varOmega}}}_{N}}g_{N} v\qquad \forall v\in {H}_{0,\partial{{{\varOmega}}}_{D}}^{1}({{{\varOmega}}}) $$

of problem (1).

We define the *broken-norm error*

17 $$  \|u-u_{h}\|_{\ast}=\left( \sum\limits_{k=1,2}\|u-u_{h}\|_{H^{1}({{{\varOmega}}}_{k})}^{2}\right)^{1/2}.  $$

Let $u_{k}^{\lambda }$ be the the weak harmonic extension of *λ* to *Ω*_*k*_, i.e. the solution of the problem

$$ {u}_{k}^{\lambda}\in V_{k}: \quad a_{k}({u}_{k}^{\lambda},v)=0 \quad \forall v\in {{V}_{k}^{0}}, \qquad {u}_{k}^{\lambda}=\lambda ~\text{ on }{{{\varGamma}}}, $$

and $\widehat {u}_{k}$ be the solution in *Ω*_*k*_ of the elliptic problem with null trace on the interface, i.e.

$$ {\widehat{u}}_{k}\in {V_{k}^{0}}: \quad a_{k}({\widehat{u}}_{k},v)=(f,v)_{L^{2}({{{\varOmega}}}_{k})} \qquad \forall v\in {V_{k}^{0}}. $$

Thanks to the linearity of the problem, when *λ* = *u*_|*Γ*_ we have $u_{k}=u_{k}^{\lambda }+{\widehat {u}}_{k}$ for *k* = 1,2.

The following convergence result has been proved in [16, Theorems 8 and 10] in the case that *Γ* is a flat interface and the interpolation operators *π*_12_ and *π*_21_ are based on the Lagrange interpolation.

#### **Theorem 1**

Assume that the weak solution *u* of the monodomain elliptic problem belongs to *H*^*s*^(*Ω*), for some *s* > 3/2, that *λ* = *u*_|*Γ*_∈ *H*^*σ*^(*Γ*) for some *σ* > 1 and that $r_{2}=\partial _{L_{2}} u_{2}\in H^{\tau }({{{\varGamma }}})$ for some *τ* > 0. Then there exist *q* ∈ [1/2,1[, *z* ∈ [3/2,2[, and a constant *c* > 0 independent of both *h*_1_ and *h*_2_ s.t.

18 $$ \begin{array}{@{}rcl@{}} \|u-u_{h}\|_{\ast} &\leq& c \left\{\left( h_{1}^{{{\varrho}}_{1}-1/2}\left( 1+\left( h_{2}/h_{1}\right)^{q}\right)+h_{2}^{{{\varrho}}_{2}-1/2}\right) \|\lambda\|_{H^{\sigma}({{{\varGamma}}})}\right.\\ && + \sum\limits_{k=1,2} h_{k}^{\ell_{k}-1}\left( \|u_{k}\|_{H^{s}({{{\varOmega}}}_{k})} + u_{k}^{\lambda}\|_{H^{s}({{{\varOmega}}}_{k})} + \|{\widehat{u}}_{k}\|_{H^{s}({{{\varOmega}}}_{k})}\right)\\ && + \left.\left[\alpha h_{1}^{\zeta_{1}+1/2}+\left( 1+\left( h_{1}/h_{2}\right)^{z}\right) h_{2}^{\zeta_{2}+1/2}\right] \|{r}_{2}\|_{H^{\tau}({{{\varGamma}}})}\right\}, \end{array} $$

where, for *k* = 1,2, $\ell _{k}=\min \limits (s,p_{k}+1)$, ${{\varrho }}_{k}=\min \limits (\sigma ,p_{k}+1)$, $\zeta _{k}=\min \limits (\tau ,p_{k}+1)$, *α* = 1 if *τ* > 1 and *α* = 0 otherwise.

Moreover, denoting by E the right-hand side of (18), Theorem 8 of [16] ensures that

19 $$  \sum\limits_{k=1,2}\|\lambda-\lambda_{k,h}\|_{H^{1/2}({{{\varGamma}}})}+\|r_{2}-r_{2,h}\|_{H^{-1/2}({{{\varGamma}}})}\leq {\textsf{E}},  $$

where *λ*_*k*, *h*_ = *u*_*k*, *h*_|_*Γ*_ are the traces on *Γ*_*k*_ of the discrete (non-conforming) solution.

When the the ratio *h*_2_/*h*_1_ is uniformly bounded from below and above (i.e., the two mesh-sizes *h*_2_ and *h*_1_ vanish with the same rate), this result guarantees that the Internodes method exhibits *optimal accuracy*, i.e., the broken-norm error behaves like the maximum between the energy-norm local errors in the two subdomains.

Indeed, by applying standard trace results on polyhedral domains (see, e.g., [21, Theorem 1.4.1]), we have that $\lambda \in H^{s-\frac {1}{2}}({{{\varGamma }}})$ and $r_{2}\in H^{s-\frac {3}{2}}({{{\varGamma }}})$ and we can take $\sigma =s-\frac {1}{2}$ and $\tau =s-\frac {3}{2}$ in (18). It follows that $\rho _{k} = \min \limits \{s-\frac {1}{2},p_{k}+1\}$ and $\zeta _{k}=\min \limits \{s-\frac {3}{2},p_{k}+1\}$ and the minimum exponent among *ℓ*_*k*_ − 1, $\rho _{k}-\frac {1}{2}$, and $\zeta _{k}+\frac {1}{2}$ is *ℓ*_*k*_ − 1.

Denoting by *C* = *C*(*u*) a positive constant depending on the exact solution *u* (and then also on the trace *λ* and the conormal derivative *r*_2_) but independent of *h*_1_ and *h*_2_, and recalling that we take *h*_2_/*h*_1_ uniformly bounded from below and above, we have

$$ \begin{array}{@{}rcl@{}} {\textsf{E}} &\leq& C \sum\limits_{k=1,2}\left( h_{k}^{\rho_{k}-\frac{1}{2}}+ h_{k}^{\ell_{k}-1}+h_{k}^{\zeta_{k}+\frac{1}{2}}\right)\\ &\leq& C \sum\limits_{k=1,2}h_{k}^{\ell_{k}-1}. \end{array} $$

Thus, (18) and (19) can be summarized as

20 $$  \|u-u_{h}\|_{\ast} + \sum\limits_{k=1,2}\|\lambda-\lambda_{k,h}\|_{H^{1/2}({{{\varGamma}}})}+\|r_{2}-r_{2,h}\|_{H^{-1/2}({{{\varGamma}}})} \leq C \left( h_{1}^{\ell_{1}-1}+h_{2}^{\ell_{2}-1}\right).  $$

#### *Remark 3*

Theorem 1 expresses the convergence order of the Internodes method with respect to the mesh sizes *h*_1_ and *h*_2_, but not with respect to the local polynomial degree *p*. The convergence with respect to *p* is showed numerically in Section 5, its analysis is in progress.

## Conservation Properties

Let us consider the balance conditions (5)–(8). We are interested in analyzing the discrete counterparts of *TF*, *TW*, *TF*_*a*_ and *TW*_*a*_ in order to measure the conservation properties of a non-conforming domain decomposition method like Internodes. As a matter of fact, it is not guaranteed that all such discrete counterparts are null, even when the meshes are conforming on *Γ* and the polynomial degrees coincide in the two subdomains.

**Fig. 2:**
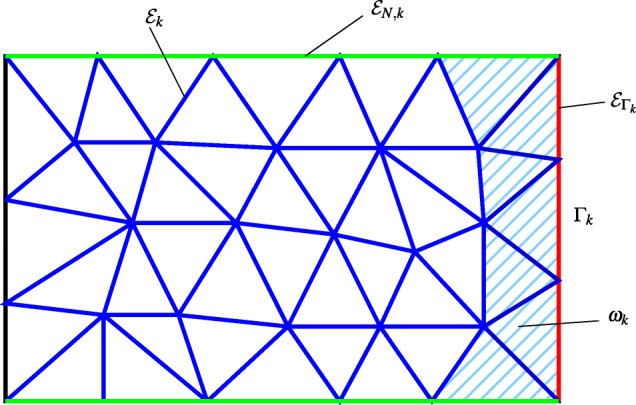
The triangulation in *Ω*_*k*_ and the sets *ω*_*k*_ (light blue region), $\mathcal {E}_{k}$ (blue edges), $\mathcal {E}_{k,N}$ (green edges), and $\mathcal {E}_{{{{\varGamma }}}_{k}}$ (red edges)

Let $\mathcal {E}_{{{{\varGamma }}}_{k}}$ be the set of the edges of the elements in $\mathcal {T}_{k,h}$ that belong to *Γ*_*k*_ (see Fig. 2) and define

$$ \begin{array}{@{}rcl@{}} TF_{h} &=& \sum\limits_{e\in \mathcal{E}_{{{{\varGamma}}}_{1}}}{\int}_{e} \nu\frac{\partial u_{1,h}}{\partial\textbf{n}_{1}} + \sum\limits_{e\in \mathcal{E}_{{{{\varGamma}}}_{2}}}{\int}_{e} \nu\frac{\partial u_{2,h}}{\partial\textbf{n}_{2}}, \\ TW_{h} &=& \sum\limits_{e\in \mathcal{E}_{{{{\varGamma}}}_{1}}}{\int}_{e} \nu\frac{\partial u_{1,h}}{\partial\textbf{n}_{1}}   u_{1,h} + \sum\limits_{e\in \mathcal{E}_{{{{\varGamma}}}_{2}}}{\int}_{e} \nu\frac{\partial u_{2,h}}{\partial\textbf{n}_{2}}  u_{2,h}, \\ TF_{a,h} &=& \sum\limits_{k=1,2}\left[a_{k}(u_{k,h},\overline{\mathcal{R}}_{k}1) - \mathcal{F}_{k}(\overline{\mathcal{R}}_{k}1)\right], \\ TW_{a,h} &=& \sum\limits_{k=1,2}\left[a_{k}(u_{k,h},\overline{u}_{k}) - \mathcal{F}_{k}(\overline{u}_{k})\right], \end{array} $$

where $\overline {u}_{k}=\overline {\mathcal {R}}_{k}({u_{k,h}}|_{{{{\varGamma }}}_{k}})$ is the finite-element extension of $u_{k,h}|_{{{{\varGamma }}}_{k}}$ to *Ω*_*k*_.

Although the identities *TF* = *TF*_*a*_ and *TW* = *TW*_*a*_ are guaranteed at the continuous level, analogous identities are no longer valid at the discrete level. Indeed, by counter-integrating by parts both *TF*_*a*, *h*_ and *TW*_*a*, *h*_, we obtain

$$ TF_{a,h}=TF_{h} + \sum\limits_{k=1,2}B_{k}(u_{k,h}, \overline{\mathcal{R}}_{k} 1), \quad TW_{a,h}=TW_{h} + \sum\limits_{k=1,2}B_{k}(u_{k,h},\overline{u}_{k}), $$

where

21 $$ \begin{array}{@{}rcl@{}} B_{k}(u_{k,h},\overline{\mu}_{k}) &=& \sum\limits_{T\in \omega_{k}}{\int}_{T}(Lu_{k,h}-f) \overline{\mu}_{k} + \sum\limits_{e\in\mathcal{E}_{k}}{\int}_{e} [\![\nu\nabla u_{k,h}]\!]\overline{\mu}_{k}\\ &&+\sum\limits_{e\in \mathcal{E}_{k,N}}{\int}_{e} \left( \nu\frac{\partial u_{k,h}}{\partial\textbf{n}_{k}}-g_{N}\right)\overline{\mu}_{k}, \end{array} $$

$\overline {\mu }_{k}=\overline {\mathcal {R}}_{k} \mu _{k,h}$ is the finite-dimensional extension to *Ω*_*k*_ of any *μ*_*k*, *h*_ ∈ *Y*_*k*, *h*_, [ [**w**] ] = **w**^+^ ⋅**n**^+^ + **w**^−^⋅**n**^−^ (following the standard notation of Discontinuous Galerkin methods), $\mathcal {E}_{k,N}$ is the set of the edges of the elements in $\mathcal {T}_{k,h}$ that belong to *∂Ω*_*k*, *N*_, *ω*_*k*_ is the set of elements in $\mathcal {T}_{k,h}$ having an edge on *Γ*_*k*_, and $\mathcal {E}_{k}$ is the set of the edges internal to *Ω*_*k*_ (see Fig. 2). Notice that the finite element extension $\overline {\mathcal {R}}_{k} \mu _{k,h}$ is null on the blue edges that are not internal to *ω*_*k*_.

### Algebraic Form of *TF*_*a*, *h*_ and *TW*_*a*, *h*_

The terms *TF*_*a*, *h*_ and *TW*_*a*, *h*_ are strictly connected with the residual arrays $\textbf {r}^{(k)}_{{{{\varGamma }}}}$ introduced in Section 3, and they can be easily computed by algebraic operations as follows [10]. We denote by **1**^(*k*)^ the array of size *n*_*k*_ whose entries are all equal to 1. It holds:

$$ \begin{array}{@{}rcl@{}} (\textbf{1}^{(k)})^{T} {\textbf{r}}_{{{{\varGamma}}}}^{(k)} &=& \sum\limits_{i=1}^{n_{k}} r_{k,i}= a_{k}(u_{k,h}, \overline{\mathcal{R}}_{k} 1)-\mathcal{F}_{k}(\overline{\mathcal{R}}_{k} 1),\\ ({\textbf{u}}_{{{{\varGamma}}}}^{(k)})^{T} \textbf{r}^{(k)}_{{{{\varGamma}}}} &=& \sum\limits_{i=1}^{n_{k}} u_{{{{\varGamma}}}_{k},i}r_{k,i} = a_{k}(u_{k,h}, \overline{u}_{k}) - \mathcal{F}_{k}(\overline{u}_{k}), \end{array} $$

where we have exploited definition (11), the fact that the Lagrange basis functions $\mu ^{(k)}_{i}$ of *Y*_*k*, *h*_ satisfy the unity partition property, i.e., ${\sum }_{i=1}^{n_{k}} \mu ^{(k)}_{i}\equiv 1$, and that $\overline {u}_{k}=\overline {\mathcal {R}}_{k}({u_{k,h}}|_{{{{\varGamma }}}_{k}})$.

It follows that

$$ TF_{a,h}=(\textbf{1}^{(1)})^{T} {\textbf{r}}_{{{{\varGamma}}}}^{(1)} +(\textbf{1}^{(2)})^{T} {\textbf{r}}_{{{{\varGamma}}}}^{(2)}, \qquad TW_{a,h}= ({\textbf{u}}_{{{{\varGamma}}}}^{(1)})^{T} {\textbf{r}}_{{{{\varGamma}}}}^{(1)} +({\textbf{u}}_{{{{\varGamma}}}}^{(2)})^{T} {\textbf{r}}_{{{{\varGamma}}}}^{(2)}. $$

Thus, it turns out very convenient to measure the conservation properties of a method by evaluating *TF*_*a*, *h*_ and *TW*_*a*, *h*_ by using these algebraic relations.

#### The Mortar Method

By adopting similar notations used to write the algebraic form (15) of the Internodes method, we write the algebraic counterpart of the Mortar method, which, instead to interpolate the trace and the normal derivative at the interface, is based on a projection process.

To this aim, let us denote by $\widetilde {P}$ the matrix implementing the projection of the trace from the interface *Γ*_1_ to *Γ*_2_. Then we notice that Mortar is a symmetric method, in the sense that the operator used to move from *Γ*_2_ to *Γ*_1_ is the transposed operator of $\widetilde {P}$.

The algebraic form of Mortar reads:

22 $$  \left\{ \begin{array}{ll} {A}_{II}^{(k)} {\textbf{u}}_{I}^{(k)}+{A}_{I{{{\varGamma}}}}^{(k)} {\textbf{u}}_{{{{\varGamma}}}}^{(k)}={\textbf{f}}_{I}^{(k)} & \text{ for }k=1,2,\\ {\textbf{u}}_{{{{\varGamma}}}}^{(2)}=\widetilde{P}{\textbf{u}}_{{{{\varGamma}}}}^{(1)},\\ {\textbf{r}}_{{{{\varGamma}}}}^{(1)}+\widetilde{P}^{T}{\textbf{r}}_{{{{\varGamma}}}}^{(2)} =\textbf{0}. \end{array}\right.  $$

Since the projection matrix $\widetilde {P}$ satisfies the property $\widetilde {P} \textbf {1}_{1}=\textbf {1}_{2}$ (this means that a constant function on *Γ*_1_ is mapped on the same constant function on *Γ*_2_), the identities

23 $$  TF_{a,h} = \sum\limits_{k=1,2}(\textbf{1}^{(k)})^{T} \textbf{r}^{(k)}_{{{{\varGamma}}}}=0 \qquad\text{ and } \qquad TW_{a,h}=\sum\limits_{k=1,2}(\textbf{u}^{(k)})^{T} \textbf{r}^{(k)}_{{{{\varGamma}}}} = 0  $$

immediately follow from (22)_2,3_.

#### The Internodes Method

On the contrary the Internodes method is not symmetric, since the two intergrid matrices *P*_21_ and $M_{{{{\varGamma }}}_{1}}P_{21}M_{{{{\varGamma }}}_{2}}^{-1}$ are not one the transpose of the other, thus there is no way that (23) are exactly satisfied by Internodes, but in the next Section we will prove that both *TF*_*a*, *h*_ ans *TW*_*a*, *h*_ provided by Internodes go to zero like the broken-norm error when *h*_1_, *h*_2_ tend to zero with the same rate. Clearly this result is weaker than (23), nevertheless we know that the broken-norm error of the Internodes method behaves like that of the Mortar method [16], and numerical results (see Section 5) show that *TF*_*h*_ and *TW*_*h*_ behave in the same manner for Internodes and Mortar methods.

Thus the question is: *Are* (23) *necessary and sufficient conditions to guarantee that a method is conservative and, not less important, accurate?*

We state that *TF*_*a*, *h*_ = 0 and *TW*_*a*, *h*_ = 0 alone do not guarantee that the coupling method one is using is convergent. This is the case of the Weighted Average Continuity Approach (WACA) proposed in [10, Sect. 3.5].

#### WACA

WACA can be formulated like (22), but with the matrix $\widetilde {P}$ replaced by $M_{{{{\varGamma }}}_{2}}^{-1}S_{2}P_{21}S_{1}^{-1}M_{{{{\varGamma }}}_{1}}$, where $M_{{{{\varGamma }}}_{k}}$ are the interface mass matrices defined in (13), *P*_21_ is the interpolation matrix associated with the interpolation operator *π*_21_ (see (9)), while *S*_*k*_ is the lumped interface mass matrix^1^ on *Γ*_*k*_. In view of the symmetry of WACA (like for Mortar), the identities (23) are satisfied.

However, when the discretization in almost one subdomain is based on ${\mathbb {Q}}_{p}$ Spectral Element Methods with Numerical Integration (SEM–NI), in virtue of the fact the SEM–NI mass matrix is diagonal, we have that $S_{k}=M_{{{{\varGamma }}}_{k}}$ and $\widetilde {P}=P_{21}$, so that WACA method coincides with the so-called *pointwise matching* method that was presented in the seminal mortar paper [6, eqs. (3.5)–(3.7)] and that is notoriously sub-optimal, as proven in [6, Sect. 3.2] and numerically corroborated in [2] for spectral elements discretizations (see also Fig. 5).

### Analysis of Conservation Properties

So far, set $h=\max \limits \{h_{1},h_{2}\}$ and let us give the following definition of conservation for a multidomain approach.

#### **Definition 1**

A multidomain approach is *conservative* at least of order *q* with respect to *h* if |*TF*_*h*_|, |*TW*_*h*_|, |*TF*_*a*, *h*_| and |*TW*_*a*, *h*_| are $\mathcal {O}(h^{q})$ when *h* tends to zero.^2^

#### Analysis of Conservation Properties of the Internodes Method

The following theorems ensure that the discrete total force *TF*_*h*_ and *TF*_*a*, *h*_ and the discrete total work *TW*_*h*_ and *TW*_*a*, *h*_ converge to zero as optimally as the broken-norm error (20) and then, that the Internodes method is conservative of the same order of the broken-norm error.

In the whole Section make the following assumption.

##### **Assumption 2**

Let us assume that the discretisation of the interface *Γ* is geometrically conforming, i.e., $\cup _{e\in \mathcal {E}_{{{{\varGamma }}}_{1}}} e = \cup _{e\in \mathcal {E}_{{{{\varGamma }}}_{2}}} e = {{{\varGamma }}}$ and that the intergrid operators *π*_12_ and *π*_21_ are based on the Lagrange interpolation. We also assume that there exist two positive constants *c*_1_ and *c*_2_ such that *c*_1_ ≤ *h*_1_/*h*_2_ ≤ *c*_2_ when *h*_1_, *h*_2_ → 0.

##### *Remark 4*

When the interfaces are not geometrically conforming, we have two difficulties in analysing the conservation properties: first we should be able to quantify the non-conformity of geometric type (and this is not often possible), second we should have convergence estimates of the Internodes method when RBF interpolation instead of Lagrange interpolation is used, and we do not have it. We notice that, for high polynomial degree *p* (typically when *p* ≥ 5), the RBF interpolation does not always exploit the same convergence order of the Lagrange interpolation and this can downgrade the accuracy of the Internodes method.

##### **Theorem 2**

Let Assumptions 1 and 2 be satisfied. If *u* ∈ *H*^*s*^(*Ω*) with *s* > 3/2, then there exists a positive constant *C* depending on the data (the domain and the coefficient functions) and on the exact solution *u*, but independent of *h*_1_ and *h*_2_ such that^3^

$$ |TF_{h}|\leq C\left( h_{1}^{\ell_{1}-1}+h_{2}^{\ell_{2}-1}\right), \quad \text{ and }\quad |TW_{h}|\leq C\left( h_{1}^{\ell_{1}-1}+h_{2}^{\ell_{2}-1}\right), $$

where, for *k* = 1,2, $\ell _{k}=\min \limits (s,p_{k}+1)$, where *p*_*k*_ is the polynomial degree in the domain *Ω*_*k*_.

##### *Proof*

First we analyse *TF*_*h*_. Since $\cup _{e\in \mathcal {E}_{{{{\varGamma }}}_{k}}} e$ for *k* = 1,2, and **n**_1_ = −**n**_2_, we have

$$ \begin{array}{@{}rcl@{}} TF_{h} &=& \sum\limits_{e\in \mathcal{E}_{{{{\varGamma}}}_{1}}}{\int}_{e} \nu\frac{\partial u_{1,h}}{\partial\textbf{n}_{1}}+ \sum\limits_{e\in \mathcal{E}_{{{{\varGamma}}}_{2}}}{\int}_{e} \nu\frac{\partial u_{2,h}}{\partial\textbf{n}_{2}}\\ &=& {\int}_{{{{\varGamma}}}} \nu\frac{\partial u_{1,h}}{\partial\textbf{n}_{1}} + {\int}_{{{{\varGamma}}}} \nu\frac{\partial u_{2,h}}{\partial\textbf{n}_{2}} \\ &=& {\int}_{{{{\varGamma}}}} \nu\frac{\partial u_{1,h}}{\partial\textbf{n}_{1}} - {\int}_{{{{\varGamma}}}} \nu\frac{\partial u}{\partial\textbf{n}_{1}} - {\int}_{{{{\varGamma}}}} \nu\frac{\partial u}{\partial\textbf{n}_{2}} + {\int}_{{{{\varGamma}}}} \nu\frac{\partial u_{2,h}}{\partial\textbf{n}_{2}}\\ &=& {\int}_{{{{\varGamma}}}} \nu\frac{\partial \left( u_{1,h} - u\right)}{\partial\textbf{n}_{1}} + {\int}_{{{{\varGamma}}}} \nu\frac{\partial \left( u_{2,h} - u\right) }{\partial\textbf{n}_{2}}. \end{array} $$

From Cauchy–Schwarz inequality and the trace theorem for polygons and polyhedra [21, 27], we have that

$$ \begin{array}{@{}rcl@{}} |TF_{h}| &\leq& \|\nu\|_{L^{\infty}({{{\varOmega}}})} |{{{\varGamma}}}| \sum\limits_{k=1,2} \left\|\frac{\partial \left( u_{k,h} - u\right)}{\partial\textbf{n}_{k}} \right\|_{H^{-1/2}({{{\varGamma}}})}\\ &\leq& C \sum\limits_{k=1,2} \|u_{k,h} - u\|_{H^{1}({{{\varOmega}}}_{k})}\leq C {\textsf{E}}. \end{array} $$

The proof for *TW*_*h*_ needs few more steps and is based on similar arguments:

$$ \begin{array}{@{}rcl@{}} TW_{h} &=& \sum\limits_{e\in \mathcal{E}_{{{{\varGamma}}}_{1}}}{\int}_{e} \nu\frac{\partial u_{1,h}}{\partial\textbf{n}_{1}}   u_{1,h} + \sum\limits_{e\in \mathcal{E}_{{{{\varGamma}}}_{2}}}{\int}_{e} \nu\frac{\partial u_{2,h}}{\partial\textbf{n}_{2}}  u_{2,h}\\ &=& {\int}_{{{{\varGamma}}}} \nu\frac{\partial u_{1,h}}{\partial\textbf{n}_{1}}   u_{1,h} + {\int}_{{{{\varGamma}}}} \nu\frac{\partial u_{2,h}}{\partial\textbf{n}_{2}}  u_{2,h} - {\int}_{{{{\varGamma}}}} \nu\frac{\partial u}{\partial\textbf{n}_{1}} u - {\int}_{{{{\varGamma}}}} \nu\frac{\partial u}{\partial\textbf{n}_{2}}  u \\ &=& \sum\limits_{k=1,2}\left[ {\int}_{{{{\varGamma}}}} \nu\frac{\partial u_{k,h}}{\partial\textbf{n}_{k}}   u_{k,h} -{\int}_{{{{\varGamma}}}} \nu\frac{\partial u}{\partial\textbf{n}_{k}}   u_{k,h} +{\int}_{{{{\varGamma}}}} \nu\frac{\partial u}{\partial\textbf{n}_{k}}   u_{k,h} - {\int}_{{{{\varGamma}}}} \nu\frac{\partial u}{\partial\textbf{n}_{k}}   u \right]\\ &=& \sum\limits_{k=1,2}\left[ {\int}_{{{{\varGamma}}}} \nu\left( \frac{\partial u_{k,h}}{\partial\textbf{n}_{k}} - \frac{\partial u}{\partial\textbf{n}_{k}} \right)  u_{k,h} +{\int}_{{{{\varGamma}}}} \nu\frac{\partial u}{\partial\textbf{n}_{k}} \left( u_{k,h} - u \right)\right]. \end{array} $$

Thanks to the trace theorem for Sobolev spaces and the triangle inequality, we also have that

24 $$ \begin{array}{@{}rcl@{}} \|u_{k,h}\|_{H^{1/2}({{{\varGamma}}})} &\leq& c\|u_{k,h}\|_{H^{1}({{{\varOmega}}}_{k})} \leq c\left( \|u_{k,h} - u|_{{{{\varOmega}}}_{k}}\|_{H^{1}({{{\varOmega}}}_{k})} + \|u|_{{{{\varOmega}}}_{k}}\|_{H^{1}({{{\varOmega}}}_{k})}\right)\\ &\leq& (c h_{k}^{\ell_{k}-1} + 1) \|u|_{{{{\varOmega}}}_{k}}\|_{H^{1}({{{\varOmega}}}_{k})} \leq (c h_{k}^{\ell_{k}-1} + 1)\|u\|_{H^{1/2}({{{\varGamma}}})}, \end{array} $$

where *c* is a positive constant depending on *Ω*_*k*_ but independent of *u*. Then, recalling that *λ* = *u*|_*Γ*_ and *λ*_*k*, *h*_ = (*u*_*k*, *h*_)|_*Γ*_, exploiting again the trace inequality and the estimate (18), when *h*_1_, *h*_2_ → 0 we get

$$ \begin{array}{@{}rcl@{}} |TW_{h}| &\leq& C\sum\limits_{k=1,2}\left[ \left\|\frac{\partial\left( u_{k,h} - u\right)}{\partial\textbf{n}_{k}} \right\|_{H^{-1/2}({{{\varGamma}}})} \|u\|_{H^{1/2}({{{\varGamma}}})}\right.\\ && \qquad\quad \left. + \left\|\frac{\partial u}{\partial \textbf{n}_{k}}\right\|_{H^{-1/2}({{{\varGamma}}})} \|\lambda_{k,h} - \lambda_{k}\|_{H^{1/2}({{{\varGamma}}})}\right]\\ &\leq& C\sum\limits_{k=1,2}\left[ \| u_{k,h} - u\|_{H^{1}({{{\varOmega}}}_{k})} \|u\|_{H^{1/2}({{{\varGamma}}})} + \left\|\frac{\partial u}{\partial \textbf{n}_{k}}\right\|_{H^{-1/2}({{{\varGamma}}})} \|u_{k,h}-u \|_{H^{1}({{{\varOmega}}})}\right]\\ &\leq & C  {\textsf{E}}. \end{array} $$

The proof is completed. □

Now we are going to analyse the terms *TF*_*a*, *h*_ and *TW*_*a*, *h*_. To this aim, we exploit the results proved in [16] after reformulating the problem (4) like a three fields problem as follows.

Let *λ*_1_ and *λ*_2_ ∈*Λ* represent the (a-priori) different traces of *u*_1_ and *u*_2_ on *Γ* and $r_{2}\in {{{\varLambda }}}^{\prime }$ the conormal derivative $\frac {\nu \partial u_{2}}{\partial \textbf {n}_{2}}$ on *Γ*, then problem (4) is equivalent to looking for *λ*_1_ ∈*Λ*, *λ*_2_ ∈*Λ*, and ${r}_{2}\in {{{\varLambda }}}^{\prime }$ s.t. (see [16, Theorem 2])

25 $$  \left\{\begin{array}{ll} \displaystyle\sum\limits_{k=1,2} a_{k} (u_{k}^{\lambda_{k}},\mathcal{R}_{k}\mu_{k}) + \langle {r}_{2},\mu_{1}- \mu_{2}\rangle = \sum\limits_{k=1,2} [(f,\mathcal{R}_{k}\mu_{k})_{L^{2}({{{\varOmega}}}_{k})} -a_{k}({\widehat{u}}_{k},\mathcal{R}_{k}\mu_{k})] \\ {\kern214pt} \forall (\mu_{1},\mu_{2})\in {{{\varLambda}}}\times{{{\varLambda}}},\\ \langle {t},\lambda_{1}-\lambda_{2}\rangle=0 {\kern151pt} \forall {t}\in {{{\varLambda}}}^{\prime}, \end{array}\right.  $$

where 〈⋅,⋅〉 denotes the duality between *Λ* and ${{{\varLambda }}}^{\prime }$.

Similarly (see [16, Theorem 3]), the Internodes problem (10) can be written in an equivalent formulation with 3 fields *λ*_1,*h*_ ∈*Λ*_1,*h*_, *λ*_2,*h*_ ∈*Λ*_2,*h*_, and $r_{2,h}\in {{{\varLambda }}}_{2,h}^{\prime }$ that are the discrete counterparts of *λ*_1_, *λ*_2_ and *r*_2_, respectively, as follows: find *λ*_1,*h*_ ∈*Λ*_1,*h*_, *λ*_2,*h*_ ∈*Λ*_2,*h*_, and ${r}_{2,h}\in {{{\varLambda }}}_{2,h}^{\prime }$ s.t.

26 $$  \left\{\begin{array}{r} \displaystyle\sum\limits_{k=1,2} a_{k}(\overline{\mathcal{H}}_{k}\lambda_{k,h}, \overline{\mathcal{R}}_{k} \mu_{k,h}) +\langle {{{\varPi}}}_{12}{r}_{2,h},\mu_{1,h}\rangle - \langle {r}_{2,h},\mu_{2,h}\rangle\\ = \displaystyle\sum\limits_{k=1,2} \left[(f,\overline{\mathcal{R}}_{k} \mu_{k,h})_{L^{2}({{{\varOmega}}}_{k})}- a_{k}({\widehat U}_{k}, \overline{\mathcal{R}}_{k} \mu_{k,h})\right]\\ {\forall (\mu_{1,h},\mu_{2,h})\in{{{\varLambda}}}_{1,h}\times{{{\varLambda}}}_{2,h},}\\ \langle t_{2,h},\lambda_{2,h} - {{{\varPi}}}_{21}\lambda_{1,h}\rangle=0 {\kern72pt} { \forall {t}_{2,h}\in {{{\varLambda}}}_{2,h}^{\prime},} \end{array}\right.  $$

where $\overline {{\mathscr{H}}}_{k}\lambda _{k,h}$ is the discrete counterpart of $u_{k}^{\lambda }$ and ${\widehat U}_{k}$ is the discrete counterpart of $\widehat {u}_{k}$.

For any (*μ*_1,*h*_, *μ*_2,*h*_) ∈*Λ*_1,*h*_ ×*Λ*_2,*h*_, we define

$$ T_{a,h}(\mu_{1,h},\mu_{2,h})=\sum\limits_{k=1,2}\left[a_{k}(u_{k,h}, \overline{\mathcal{R}}_{k}\mu_{k,h})-{\mathcal{F}}_{k}(\overline{\mathcal{R}}_{k}\mu_{k,h})\right] $$

and, in view of (26)_1_ and the fact that $u_{k,h}=\overline {{\mathscr{H}}}_{k}\lambda _{k,h}+{\widehat U}_{k}$, it holds

$$ T_{a,h}(\mu_{1,h},\mu_{2,h})=\langle r_{2,h},\mu_{2,h}\rangle-\langle {{{\varPi}}}_{12}r_{2,h}, \mu_{1,h}\rangle, $$

and we have

$$ TF_{a,h}=T_{a,h}(1,1), \qquad TW_{a,h}=T_{a,h}({u_{1,h}}|_{{{{\varGamma}}}},{u_{2,h}}|_{{{{\varGamma}}}}). $$

It is useful to define also

$$ T_{a}(\mu_{1},\mu_{2})=\sum\limits_{k=1,2}\left[a_{k}(u_{k}, {\mathcal{R}}_{k}\mu_{k})-{\mathcal{F}}_{k}({\mathcal{R}}_{k}\mu_{k})\right] $$

for any (*μ*_1_, *μ*_2_) ∈*Λ*×*Λ*. In view of (25)_1_ and the fact that $u_{k}=u^{\lambda }_{k}+\widehat {u}_{k}$, we can also write

$$ T_{a}(\mu_{1},\mu_{2})=\langle r_{2}, \mu_{2}-\mu_{1}\rangle $$

and notice that

$$ TF_{a}=T_{a}(1,1), \qquad TW_{a}=T_{a}({u_{1}}|_{{{{\varGamma}}}},{u_{2}}|_{{{{\varGamma}}}}). $$

In the next theorem we are going to prove that |*TF*_*a*, *h*_| and |*TW*_*a*, *h*_| behave like the broken-norm error (20) when *h*_1_/*h*_2_ is uniformly bounded from below and above.

##### **Theorem 3**

Under the assumptions of Theorem 2, there exists a positive constant *C* depending on the data (the domain and the coefficient functions) and on the exact solution *u*, but independent of *h*_1_ and *h*_2_ such that

$$ |TF_{a,h}|\leq C\left( h_{1}^{\ell_{1}-1}+h_{2}^{\ell_{2}-1}\right), \quad \text{ and }\quad |TW_{a,h}|\leq C\left( h_{1}^{\ell_{1}-1}+h_{2}^{\ell_{2}-1}\right). $$

##### *Proof*

Recalling that *T*_*a*_(*μ*_1_, *μ*_2_) = 0 for any (*μ*_1_, *μ*_2_) ∈*Λ*×*Λ*, it holds

$$ \begin{array}{@{}rcl@{}} |T_{a,h}(\mu_{1,h},\mu_{2,h})|&=&|T_{a}(\mu_{1},\mu_{2})-T_{a,h}(\mu_{1,h},\mu_{2,h})|\\ &=& |\langle r_{2},\mu_{1}-\mu_{2}\rangle-\langle {{{\varPi}}}_{12}r_{2,h}, \mu_{1,h}\rangle +\langle r_{2,h},\mu_{2,h}\rangle|(\pm \langle r_{2},\mu_{1,h}-\mu_{2,h}\rangle)\\ &\leq & \sum\limits_{k=1,2} |\langle r_{2},\mu_{k}-\mu_{k,h}\rangle|+|\langle r_{2}-r_{2,h},\mu_{2,h}\rangle|+|\langle r_{2}-{{{\varPi}}}_{12}r_{2,h},\mu_{1,h}\rangle|. \end{array} $$

Because we are interested in bounding |*TF*_*a*, *h*_| and |*TW*_*a*, *h*_| (that is *μ*_1,*h*_ = *μ*_2,*h*_ = 1 in the first case and $\mu _{k,h}=\lambda _{k,h}={u_{k,h}}|_{{{{\varGamma }}}_{k}}$ in the second one), when *μ*_1,*h*_ = *μ*_2,*h*_ = 1 we choose *μ*_1_ = *μ*_2_ = 1, while when $\mu _{k,h}=\lambda _{k,h}={u_{k,h}}|_{{{{\varGamma }}}_{k}}$ we choose *μ*_*k*_ = *λ* = *u*|_*Γ*_. We bound each term as follows:

- First we apply the Cauchy–Schwarz inequality
  $$ \sum\limits_{k=1,2} |\langle r_{2},\mu_{k}-\mu_{k,h}\rangle|\leq \|r_{2}\|_{H^{-1/2}({{{\varGamma}}})}\left( \|\mu_{1}-\mu_{1,h}\|_{H^{1/2}({{{\varGamma}}})}+\|\mu_{2}-\mu_{2,h}\|_{H^{1/2}({{{\varGamma}}})}\right). $$
  Now, if *μ*_1,*h*_ = *μ*_2,*h*_ = 1 (and *μ*_1_ = *μ*_2_ = 1), the left-hand side of the previous formula is null; on the other hand, if $\mu _{k,h}=\lambda _{k,h}={u_{k,h}}|_{{{{\varGamma }}}_{k}}$ (and *μ*_*k*_ = *λ* = *u*|_*Γ*_), by applying (20), we have
  $$ \sum\limits_{k=1,2} |\langle r_{2},\lambda_{k}-\lambda_{k,h}\rangle|\leq C\left( h_{1}^{\ell_{1}-1}+h_{2}^{\ell_{2}-1}\right). $$
  Notice that *r*_2_ is related to the exact solution *u* and its norm is included in the constant *C*.
- By applying the Cauchy–Schwarz inequality and (20) it holds
  $$ \begin{array}{@{}rcl@{}} |\langle r_{2}-r_{2,h},\mu_{2,h}\rangle|&\leq& \|r_{2}-r_{2,h}\|_{H^{-1/2}({{{\varGamma}}})} \|\mu_{2,h}\|_{H^{1/2}({{{\varGamma}}})}\\ &\leq & C\left( h_{1}^{\ell_{1}-1}+h_{2}^{\ell_{2}-1}\right)\|\mu_{2,h}\|_{H^{1/2}({{{\varGamma}}})}. \end{array} $$
  If *μ*_2,*h*_ = 1, then $\|\mu _{2,h}\|_{H^{1/2}({{{\varGamma }}})}$ is the measure of *Γ*; while if $\mu _{2,h}={u_{2,h}}|_{{{{\varGamma }}}_{2}}$, by applying (24) we can conclude
  $$ |\langle r_{2}-r_{2,h},\mu_{2,h}\rangle|\leq C \left( h_{1}^{\ell_{1}-1}+h_{2}^{\ell_{2}-1}\right). $$
- Let $t_{2}=\pi _{h_{2}}r_{2}$ denote the *L*^2^-projection of *r*_2_ onto *Y*_2,*h*_. By applying the triangle inequality it holds
  $$ \begin{array}{@{}rcl@{}} |\langle r_{2}-{{{\varPi}}}_{12}r_{2,h},\mu_{1,h}\rangle| &\leq& |\langle r_{2}-t_{2},\mu_{1,h}\rangle|+ |\langle t_{2}-{{{\varPi}}}_{12}t_{2},\mu_{1,h}\rangle|\\ &&+ |\langle {{{\varPi}}}_{12}(t_{2}-r_{2,h}),\mu_{1,h}\rangle|. \end{array} $$

We examine each term in the right-hand side of the previous inequality:

- by Cauchy–Schwarz inequality and the projection error [16, (115)] it holds
  $$ \begin{array}{@{}rcl@{}} |\langle r_{2}-t_{2},\mu_{1,h}\rangle|&\leq& \|r_{2}-t_{2}\|_{H^{-1/2}({{{\varGamma}}})} \|\mu_{1,h}\|_{H^{1/2}({{{\varGamma}}})}\\ &\leq& c h_{2}^{\zeta_{2}+1/2}\|r_{2}\|_{H^{\tau}({{{\varGamma}}})} \|\mu_{1,h}\|_{H^{1/2}({{{\varGamma}}})}, \end{array} $$
  where *ζ*_2_ has been introduced in Theorem 1.
- by interpreting the duality 〈*t*_2_ −*π*_12_*t*_2_, *μ*_1,*h*_〉 as *L*^2^-product on *Γ* (both terms are finite dimensional) and applying the same arguments used in the proof of Theorem 10 of [16], we have
  $$ \begin{array}{@{}rcl@{}} |\langle t_{2}-{{{\varPi}}}_{12}t_{2},\mu_{1,h}\rangle|&\leq& c h_{1}^{1/2} \|t_{2}-{{{\varPi}}}_{12} t_{2}\|_{L^{2}({{{\varGamma}}})} \|\mu_{1,h}\|_{L^{2}({{{\varGamma}}})} \\ &\leq& ch_{1}^{1/2}\!\!\left( \!\!\alpha h_{1}^{\zeta_{1}+1/2} + \left( \!\!1 + \left( \frac{h_{1}}{h_{2}}\!\right)^{z}\!\right)\! h_{2}^{\zeta_{2}+1/2}\right) \|r_{2}\|_{H^{\tau}({{{\varGamma}}})}\|\mu_{1,h}\|_{L^{2}({{{\varGamma}}})}\\ &&\text{(by (18), (20), and (24))}\\ &\leq& C\left( h_{1}^{\ell_{1}-1}+h_{2}^{\ell_{2}-1}\right), \end{array} $$
  where *α* and *z* have the same meaning as in (18);
- denoting by ${{{\varPi }}}_{12}^{\ast }$ the adjoint operator of *π*_12_, by the fact that $c=\|{{{\varPi }}}_{12}^{\ast }\|=\|{{{\varPi }}}_{12}\|$ and thanks to Lemma 5 of [16], we have
  $$ \begin{array}{@{}rcl@{}} |\langle {{{\varPi}}}_{12}(\pi_{h_{2}}r_{2}-r_{2,h}),\mu_{1,h}\rangle|& =& |\langle \pi_{h_{2}}r_{2}-r_{2,h},{{{\varPi}}}_{12}^{*}\mu_{1,h}\rangle|\\ &\leq& c \|\pi_{h2}r_{2}-r_{2,h}\|_{H^{-1/2}({{{\varGamma}}})} \|\mu_{1,h}\|_{H^{1/2}({{{\varGamma}}})}. \end{array} $$
  Now we apply the triangle inequality to $\|\pi _{h2}r_{2}-r_{2,h}\|_{H^{-1/2}({{{\varGamma }}})}$:
  $$ \|\pi_{h2}r_{2}-r_{2,h}\|_{H^{-1/2}({{{\varGamma}}})} \leq \|r_{2}-r_{2,h}\|_{H^{-1/2}({{{\varGamma}}})}+\|r_{2}- \pi_{h2}r_{2}\|_{H^{-1/2}({{{\varGamma}}})} $$
  and by exploiting again the projection error [16, (115)], (20), (18) and (24), we can conclude that
  $$ \begin{array}{@{}rcl@{}} |\langle {{{\varPi}}}_{12}(\pi_{h_{2}}r_{2}-r_{2,h}),\mu_{1,h}\rangle| &\leq& C \left( h_{2}^{\zeta_{2}+1/2}\|r_{2}\|_{H^{\tau}({{{\varGamma}}})}\right. \\ &&+ h_{1}^{1/2}\left( \alpha h_{1}^{\zeta_{1}+1/2}+\left( 1+\left( \frac{h_{1}}{h_{2}}\right)^{z}\right)h_{2}^{\zeta_{2}+1/2}\right) \|r_{2}\|_{H^{\tau}({{{\varGamma}}})} \\ &&\left. + \left( h_{1}^{\ell_{1}-1}+h_{2}^{\ell_{2}-1}\right) \|\mu_{1,h}\|_{H^{1/2}({{{\varGamma}}})}\right)\\ &\leq& C\left( h_{1}^{\ell_{1}-1}+h_{2}^{\ell_{2}-1}\right). \end{array} $$

Finally, by summing up all terms the thesis follows. □

The following theorem is an immediate consequence of Theorems 2 and 3.

##### **Theorem 4**

Set $\ell =\min \limits \{\ell _{1},\ell _{2}\}$ and $h=\max \limits \{h_{1},h_{2}\}$, under the assumptions of Theorem 2, the Internodes method is conservative up to order *q* = *ℓ* − 1, that is the order of the broken-norm error.

We can also formulate an upper bound for the forms *B*_*k*_ defined in (21):

##### **Corollary 1**

Under the assumptions of Theorem 2, it holds

$$ B_{k}(u_{k,h},1) = \mathcal{O}(h^{\ell-1})\quad \text{ and }\quad B_{k}(u_{k,h},\overline{u}_{k,h})= \mathcal{O}(h^{\ell-1})\qquad\text{ for } k=1,2. $$

## Numerical Results

The numerical results of this section corroborate the theoretical results proved above, that is the Internodes method is conservative of order *q* = *ℓ* − 1 (see Theorem 4). We consider here both 2D and 3D test cases with either geometric conformity and non-conformity at the interface. The non-conforming geometric case is not covered by the theory but the numerical results are however consistent with the geometric conforming situation.

In all cases we compare the quantities |*TF*_*h*_|, |*TF*_*a*, *h*_|, |*TW*_*h*_| and |*TW*_*a*, *h*_| with the broken-norm error (see (17)) and the *L*^2^ error $\|u-u_{h}\|_{L^{2}({{{\varOmega }}})}$. Moreover, for the first test case we compare Internodes with the Mortar method in terms of accuracy and conservation properties. We show that *TF*_*h*_ and *TW*_*h*_ behave like $\mathcal {O}(h^{p})$ for both Mortar and Internodes (exactly as the broken-norm errors do); that |*TF*_*a*, *h*_| and |*TW*_*a*, *h*_| are $\mathcal {O}(h^{p})$ for Internodes and null up to the machine precision for both Mortar and WACA.

In the whole section we set $h=\max \limits \{h_{1},h_{2}\}$.

### Test Case #1: *d* = 2, Flat Interface (Geometric Conformity)

Let us set the domain *Ω* = (0,2) × (0,1) and the coefficients *ν* = *γ* = 1; the right-hand side *f* and the boundary datum *g*_*N*_ are such that the exact solution is $u(x,y)=(2x+1)(2y+1)\sin \limits (x\pi /2)\sin \limits (\pi y)$. Then we decompose *Ω* into the subdomains *Ω*_1_ = (0,1) × (0,1) and *Ω*_2_ = (1,2) × (0,1), so that the interface is *Γ* = {1}× (0,1). Neumann boundary conditions are imposed on the horizontal sides of *Ω* and homogeneous Dirichlet boundary conditions are imposed elsewhere.

**Fig. 3:**
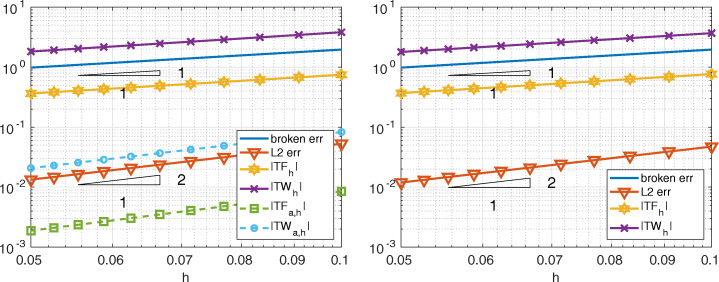
Test case #1. Internodes (left) and Mortar (right), flat interface, ${\mathbb {P}}_{1}$, finer discretization in *Ω*_1_. *TF*_*a*, *h*_ and *TW*_*a*, *h*_ are about machine precision for Mortar

In Fig. 3 we compare Internodes with the Mortar method, more precisely we consider $\mathbb {P}_{1}$ fem in both subdomains of size *h*_1_ = 1/(2*k* − 1) and *h*_2_ = 1/*k* with *k* = 10,…,20, so that the mesh is finer in the domain *Ω*_1_. The two approaches provide very similar errors (broken and *L*^2^-norms), also the quantities |*TF*_*h*_| and |*TW*_*h*_| are very similar and we have

$$ |TF_{h}|\sim |TW_{h}|\sim \|u-u_{h}\|_{\ast}= \mathcal{O}(h), \qquad \|u-u_{h}\|_{L^{2}({{{\varOmega}}})}={\mathcal{O}}(h^{2}). $$

As anticipated in Section 4, the quantities |*TF*_*a*, *h*_| and |*TW*_*a*, *h*_| behave like $\mathcal {O}(h)$ for the Internodes method while they are about the machine precision for the the Mortar method.

**Fig. 4:**
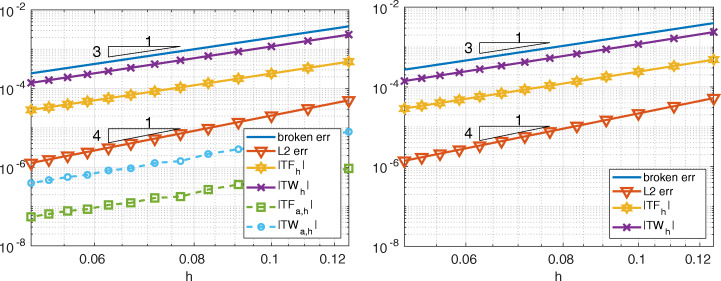
Test case #1. Internodes (left) and Mortar (right), flat interface, ${\mathbb {Q}}_{3}$, finer discretization in *Ω*_1_. *TF*_*a*, *h*_ and *TW*_*a*, *h*_ of Mortar are about machine precision

In Fig. 4 we compare Internodes with Mortar as before, but now by taking *p* = *p*_1_ = *p*_2_ = 3, thus ${\mathbb {Q}}_{3}$–SEM–NI are employed in each subdomain. Numerical results show that

$$ |TF_{h}|\sim |TW_{h}|\sim \|u-u_{h}\|_{\ast}= \mathcal{O}(h^{3}), \qquad \|u-u_{h}\|_{L^{2}({{{\varOmega}}})}={\mathcal{O}}(h^{4}). $$

The quantities |*TF*_*a*, *h*_| and |*TW*_*a*, *h*_| behave like $\mathcal {O}(h^{3})$ for the Internodes method while, again, they are about the machine precision for the Mortar method.

The different behaviour of *TF*_*a*, *h*_ and *TW*_*a*, *h*_ for the two methods strictly depends on the way the interface conditions are imposed at the interface, as we have explained in Section 4.

**Fig. 5:**
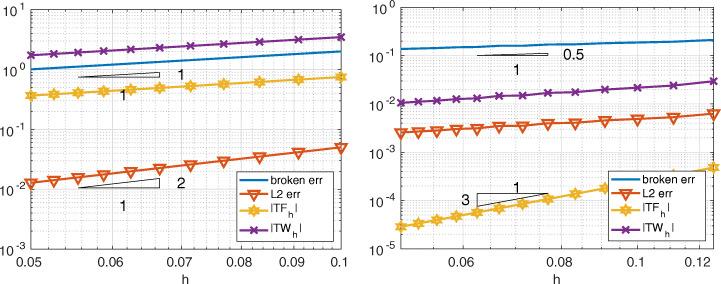
Test case #1. WACA with ${\mathbb {P}}_{1}$ (left) and ${\mathbb {Q}}_{3}$ (right) discretization, flat interface, finer discretization in *Ω*_1_. *TF*_*a*, *h*_ and *TW*_*a*, *h*_ are about machine precision

In Fig. 5, the broken-norm and the *L*^2^-norm errors, as well as *TF*_*h*_ and *TW*_*h*_, are shown for the WACA method [10]. WACA exhibits the same accuracy of Mortar and Internodes methods when ${\mathbb {P}}_{1}$ discretization is adopted in both subdomains, while it is sub-optimal for high-order spectral element discretizations.

**Fig. 6:**
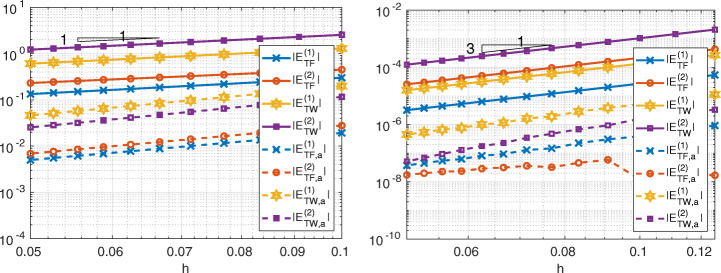
Test case #1. Internodes with ${\mathbb {P}}_{1}$ (left) and ${\mathbb {Q}}_{3}$ (right) discretization, flat interface, finer discretization in *Ω*_1_

**Fig. 7:**
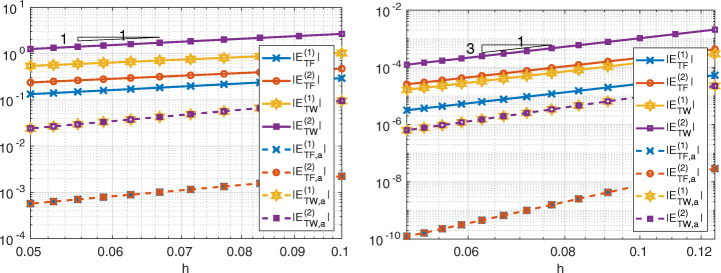
Test case #1. Mortar with ${\mathbb {P}}_{1}$ (left) and ${\mathbb {Q}}_{3}$ (right) discretization, flat interface, finer discretization in *Ω*_1_

**Fig. 8:**
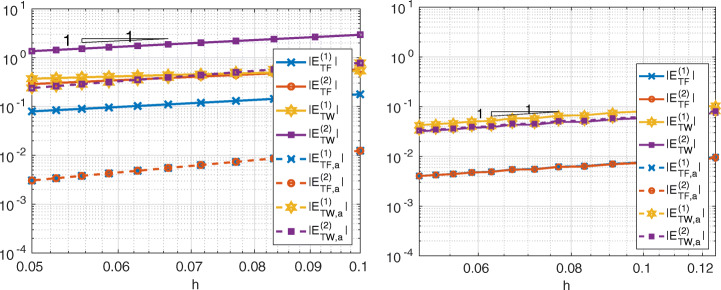
Test case #1. WACA with ${\mathbb {P}}_{1}$ (left) and ${\mathbb {Q}}_{3}$ (right) discretization, flat interface, finer discretization in *Ω*_1_

In Figs. 6, 7 and 8 we show the quantities

27 $$ \begin{array}{@{}rcl@{}} E_{TF,a}^{(k)} &=& \left|{\int}_{{{{\varGamma}}}_{k}}\frac{\partial u}{\partial \textbf{n}_{k}} -(\textbf{1}^{(k)}_{{{{\varGamma}}}})^{T}\textbf{r}^{(k)}_{{{{\varGamma}}}}\right|,\\ E_{TW,a}^{(k)} &=& \left|{\int}_{{{{\varGamma}}}_{k}}\frac{\partial u}{\partial \textbf{n}_{k}}  u -(\textbf{r}^{(k)}_{{{{\varGamma}}}})^{T}{\textbf{u}}_{{{{\varGamma}}}}^{(k)}\right|,\\ E_{TF}^{(k)} &=& \left| {\int}_{{{{\varGamma}}}_{k}} \frac{\partial u}{\partial \textbf{n}_{k}} -\sum\limits_{e\in \mathcal{E}_{{{{\varGamma}}}_{k}}}{\int}_{e} \frac{\partial u_{k,h}}{\partial\textbf{n}_{k}}\right|,\\ E_{TW}^{(k)} &=& \left|{\int}_{{{{\varGamma}}}_{k}} \frac{\partial u}{\partial \textbf{n}_{k}}  u - \sum\limits_{e\in {\mathcal{E}}_{{{{\varGamma}}}_{k}}}{\int}_{e} \frac{\partial u_{k,h}}{\partial\textbf{n}_{k}} u_{k,h}\right|  \end{array} $$

for Internodes, Mortar and WACA methods.

The values (27) measure how well the contributions from each side of the interface approximate the force and the work. We notice that for each *k* = 1,2 the quantity $(\textbf {1}^{(k)}_{{{{\varGamma }}}})^{T}\textbf {r}^{(k)}_{{{{\varGamma }}}}$ approximates ${\int \limits }_{{{{\varGamma }}}_{k}} \partial u/\partial \textbf {n}_{k}$ better than ${\sum }_{e\in {\mathcal {E}}_{{{{\varGamma }}}_{k}}}{\int \limits }_{e} \frac {\partial u_{k,h}}{\partial \textbf {n}_{k}}$ does and, similarly, $(\textbf {r}^{(k)}_{{{{\varGamma }}}})^{T}{\textbf {u}}_{{{{\varGamma }}}}^{(k)}$ approximates ${\int \limits }_{{{{\varGamma }}}_{k}} (\partial u/\partial \textbf {n}_{k})u$ better than ${\sum }_{e\in {\mathcal {E}}_{{{{\varGamma }}}_{k}}}{\int \limits }_{e} \frac {\partial u_{k,h}}{\partial \textbf {n}_{k}} u_{k,h}$ does.

Notice that *TF*_*a*, *h*_ = 0 (or *TW*_*a*, *h*_ = 0) does not imply that the single contribution $E_{TF,a}^{(k)}$ from the side *k* of the interface is approximated accurately, as in the case of the WACA method.

**Fig. 9:**
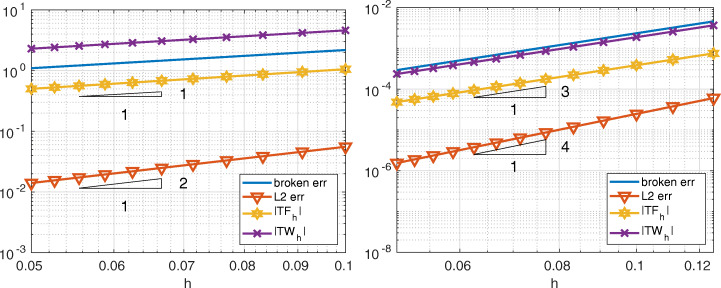
Test case #1. Conforming decomposition, with ${\mathbb {P}}_{1}$ (left) and ${\mathbb {Q}}_{3}$ (right) discretization, flat interface. *TF*_*a*, *h*_ and *TW*_*a*, *h*_ are about machine precision

Finally, in Fig. 9 we report the errors and the quantities |*TW*_*h*_|, |*TF*_*h*_| in the case of a decomposition that is conforming at the interface, i.e., with mesh-size *h* = *h*_1_ = *h*_2_ and polynomial degree *p* = *p*_1_ = *p*_2_. These pictures highlight that *TW*_*h*_ and *TF*_*h*_ are not null also in the case of conforming decomposition, without however detracting from the conservation properties of the multidomain approach; more precisely, *TW*_*h*_ and *TF*_*h*_ are $\mathcal {O}(h^{p})$ when *h*_1_, *h*_2_ → 0 uniformly. In the conforming case, *TF*_*a*, *h*_ = *TW*_*a*, *h*_ = 0 in virtue of the interface conditions (16)_2,3_. The two meshes are regular and uniform with *k* × *k* elements in each subdomain, *k* = 10,20,…,50 for ${\mathbb {P}}_{1}$ and *k* = 8,…,20 for ${\mathbb {Q}}_{3}$.

### Test Case #2: *d* = 2, Curved Interface (Geometric Non-conformity)

**Fig. 10:**
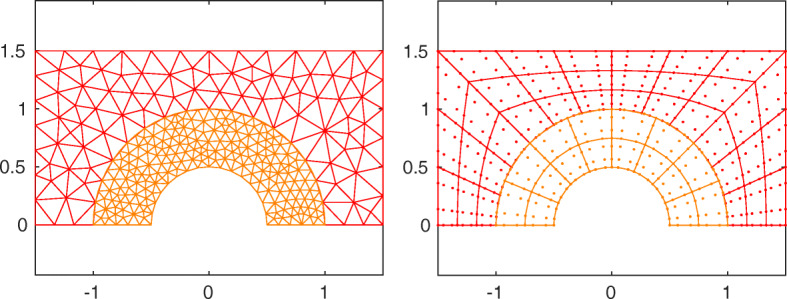
Test case #2. The non-conforming meshes (${\mathbb {P}}_{1}$ left and ${\mathbb {Q}}_{3}$ right)

Let $\mathcal {C}_{1}(0)$ be the circle of center 0 and ray 1. We set ${{{\varOmega }}}=((-1.5,1.5)\times (0,1.5))\setminus \mathcal {C}_{1}(0)$ and *ν* = *γ* = 1; moreover, *f*, *g*_*N*_ and *g*_*D*_ (*g*_*D*_ is the non-homogeneous Dirichlet datum) are such that the exact solution is $u(x,y)=\sin \limits (x\pi /2 +\pi /3)\sin \limits (\pi y+\pi /4)$. Then we impose Neumann boundary conditions on the horizontal side of the domain and Dirichlet boundary conditions elsewhere. The computational domain is split into *Ω*_2_ = {**x** ∈*Ω* : |**x**| < 1} and *Ω*_1_ = *Ω* ∖*Ω*_2_, so that the interface is a semicircle (see Fig. 10). First we consider ${\mathbb {P}}_{1}$ finite elements in both subdomains of size *h*_1_ = 1.5/*k* and *h*_2_ = 1/(*k* − 1) with *k* = 10,…,50, and then ${\mathbb {Q}}_{3}$ spectral elements in both subdomains of size *h*_1_ = 1.5/*k* and *h*_2_ = 1/(*k* − 1) with *k* = 5,…,20. The finer mesh is that in the slave domain *Ω*_2_.

**Fig. 11:**
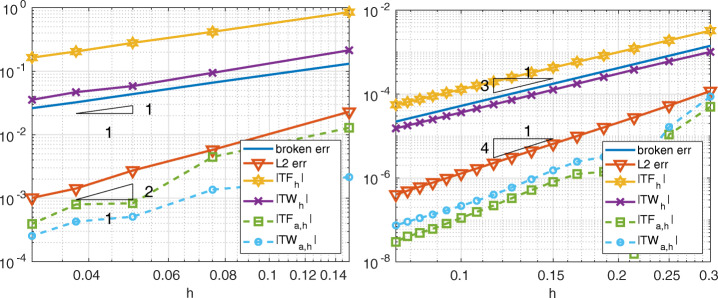
Test case #2. Internodes, curved interface, ${\mathbb {P}}_{1}$ (left) and ${\mathbb {Q}}_{3}$ (right), non-conforming discretization

For this test case we report numerical results only for the Internodes method (see Fig. 11), since, with the help of RL-RBF interpolation, Internodes does not feature any additional difficulty with respect to the case of geometric matching interfaces. On the contrary, the implementation of Mortar method for curved interfaces is far from trivial: it requires several steps such as projection, intersection, local meshing and numerical quadrature to build up the mortar interface coupling operator. These aspects are carefully addressed in [24] (see in particular Algorithm 1, Section 3.2.3).

As in the case of flat interface, all the quantities *TF*_*h*_, *TW*_*h*_, *TF*_*a*, *h*_ and *TW*_*a*, *h*_ are $\mathcal {O}(h^{p})$ (recall that $h=\max \limits \{h_{1},h_{2}\}$), ensuring the conservation properties of the Internodes method.

**Fig. 12:**
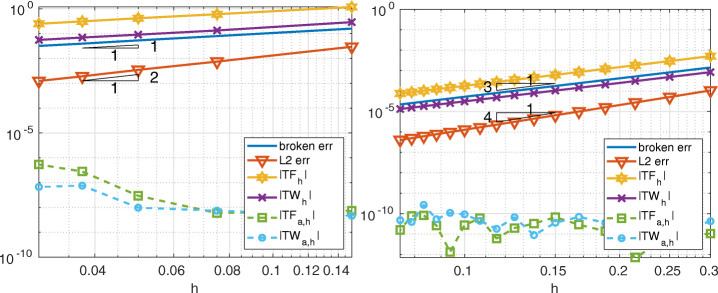
Test case #2. Internodes, curved interface, ${\mathbb {P}}_{1}$ (left) and ${\mathbb {Q}}_{3}$ (right), conforming discretization on the interface

Finally in Fig. 12 we show the errors and the quantities |*TW*_*h*_|, |*TF*_*h*_|, |*TW*_*a*, *h*_|, and |*TF*_*a*, *h*_| when the discretization at the interface is conforming; we notice that |*TW*_*a*, *h*_| and |*TF*_*a*, *h*_| are affected by rounding errors, while |*TW*_*h*_| and |*TF*_*h*_| are $\mathcal {O}(h^{p})$ as in the non-conforming case.

### Test Case #3: *d* = 2, Discontinuous Coefficients and *M* > 2 Subdomains

**Fig. 13:**
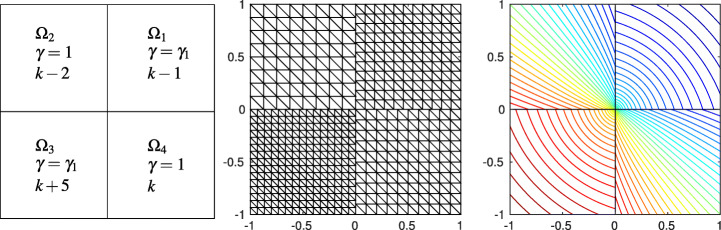
Test case # 3. The Kellogg’s test case. On the left, the decomposition of *Ω* into four subdomains. In the middle, the nonconforming ${\mathbb {P}}_{1}$ meshes for *k* = 10. On the right, the Kellogg’s solution with *α* = 0.4 and *γ*_1_ = 9.472135954999585 computed by INTERNODES and ${\mathbb {P}}_{1}$

Let us consider now the Kellogg’s test case (see, e.g., [17, 23]), in which the function *ν* is piece-wise constant and *γ* = 0. The exact solution can be written in terms of the polar coordinates *r* and *𝜃* as *u*(*r*, *𝜃*) = *r*^*α*^*μ*(*𝜃*), where *α* ∈ (0,2) is a given parameter, while *μ*(*𝜃*) is a 2*π*-periodic continuous function (more regular only when *α* = 1); see Fig. 13. When *α*≠ 1, *u* ∈ *H*^1+*α*−*ε*^(*Ω*), for any *ε* > 0; the solution features low regularity at the origin and its normal derivatives to the axis are discontinuous. The positive value *γ*_1_ depends on *α* and on two other real parameters *σ* and *ρ*. The set {*γ*_1_, *α*, *σ*, *ρ*} must satisfy a nonlinear system (see formula (5.1) of [23]). In particular we fixed *ρ* = *π*/4.

We consider here the domain splitting as well as the discretization described in [17], where the numerical convergence order of the Internodes method has been shown for different values of the parameter *α*. In [17], the convergence estimate provided by Theorem 1 for two subdomains has been confirmed, although this test case involves four subdomains instead of two.

**Fig. 14:**
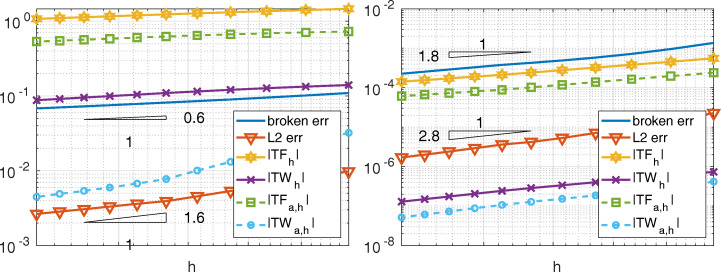
Test case #3. On the left, *α* = 0.6 and ${\mathbb {P}}_{1}$ discretization; on the right, *α* = 1.8 and ${\mathbb {Q}}_{2}$ discretization. The subdomains mesh-sizes are: *h*_1_ = 1/(*k* − 1), *h*_2_ = 1/(*k* − 2), *h*_3_ = 1/(*k* + 5) and *h*_4_ = 1/*k* in both cases

Here we show that also the conservation properties of the Internodes method are preserved when the problem features discontinuous coefficients. In Fig. 14 we compare the total force and the total work (in both strong and weak form) with the *H*^1^-broken norm error and the *L*^2^-norm error, for ${\mathbb {P}}_{1}$ (${\mathbb {Q}}_{2}$, resp) discretization in each subdomain when *α* = 0.6 (*α* = 1.8, resp.). Recalling that *u* ∈ *H*^1+*α*−*ε*^(*Ω*), it is useless to consider higher polynomial degrees.

### Test Case #4: *d* = 2, *M* > 2 Subdomains and Conservation Properties vs. *p*

This is another test case with more than two subdomains in which we show both the convergence and the conservation properties of the Internodes method with respect to the local polynomial degree *p*.

**Fig. 15:**
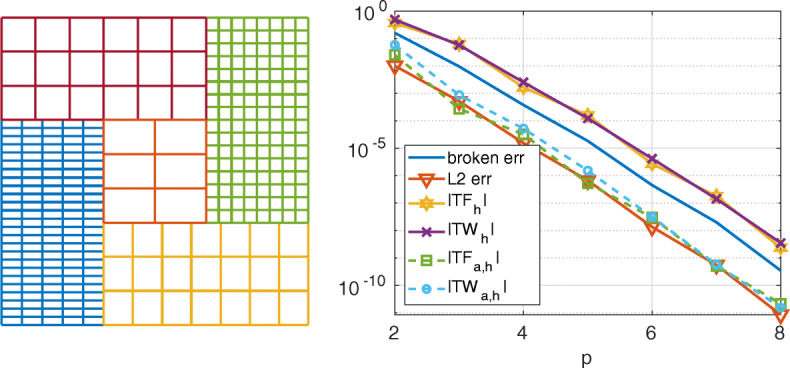
Test case #4. On the left, the domain splitting (the different colors refer to the different subdomains, the quads are the elements of the mesh, inside each element there are (*p* + 1)^2^ nodes) and the non-conforming meshes. On the right, the errors, the total forces and the total works at the interface vs. the polynomial degree *p*

The domain *Ω* = (0,3)^2^ is split in five subdomains as in a *tatami* (see Fig. 15). We have considered the same polynomial degree *p* in each subdomain, while the mesh sizes are all different, as we can evince from the left picture of Fig. 15. In the right picture of Fig. 15 we show the errors between the Internodes solution and the exact one $u(x,y)=\cos \limits ((x+y)\pi /2)(x-2y)$, as well as the weak and strong total forces and total works. The coefficients of the problem are *ν* = 1 and *γ* = 0. Dirichlet boundary conditions are imposed on the whole *∂Ω*.

The curves plotted in the right picture of Fig. 15 show that the errors (*H*^1^-broken norm and *L*^2^-norm), as well as the weak and strong total forces and total works decay more than algebraically with respect to *p*, as it is typical for spectral elements (or *hp*-fem) discretizations.

### Test Case #5: *d* = 3, Curved Interface (Geometric and Non-geometric Conformity)

Now, let *Ω* = (0,1) × (0,1) × (0,2) be split into two subdomains with curved interface ${{{\varGamma }}}_{\alpha }=\{(x,y,z)\in {\mathbb {R}}^{3}:~(x,y)\in [0,1]^{2}, z=0.3(x^{\alpha }+y^{\alpha })+1\}$, with *α* = 2 or *α* = 3. Neumann (Dirichlet, resp.) boundary conditions have been imposed on the vertical (horizontal, resp.) faces of *∂Ω*, while *f*, *g*_*D*_ and *g*_*N*_ are chosen so that the exact solution is $u(x,y,z)=\sin \limits (xyz\pi )$. The coefficients of the problem are *ν* = *γ* = 1.

**Fig. 16:**
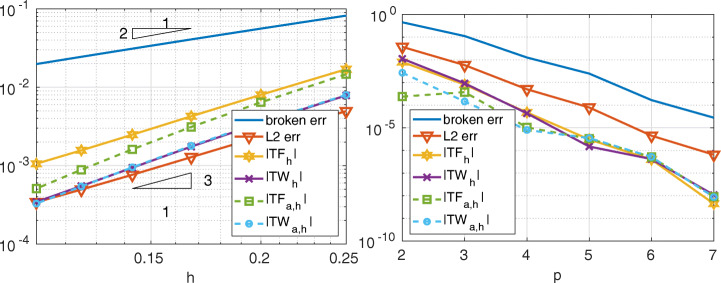
Test case #5. On the left, ${\mathbb {Q}}_{2}$ discretization, cubic interface and RBF interpolation, geometric non-conforming. On the right, ${\mathbb {Q}}_{p}$ discretization, quadratic interface and Lagrange interpolation, geometric conforming

First, we have set the interface *Γ*_*α*_ with *α* = 3 and we have considered ${\mathbb {Q}}_{2}$ discretizations in both the subdomains with variable *h*_1_ = 1/*k* and *h*_2_ = 1/(*k* + 1), for *k* = 2,…,9. RL-RBF interpolation has been used to build the interpolation matrices of Internodes, since the interface cannot be described exactly by ${\mathbb {Q}}_{2}$ isoparametric elements, thus we have geometric non-conformity. The errors, as well as the total forces and the total works are shown in the left picture of Fig. 16: they decrease at least like *h*^2^, i.e., like the *H*^1^-broken-norm error.

Then, we have set the interface *Γ*_*α*_ with *α* = 2 and we have considered ${\mathbb {Q}}_{p}$ Spectral Element discretization in both the subdomains with fixed *h*_1_ = 1/4 and *h*_2_ = 1/3, for *p* = 3,…,7. Now, Lagrange interpolation has been considered to build the intergrid matrices of Internodes. We can use Lagrange interpolation since the interface can be described exactly by ${\mathbb {Q}}_{p}$ spectral elements^4^ with *p* ≥ 2, thus the interfaces are geometric conforming. The total forces and the total works are shown in the right picture of Fig. 16: they decrease versus *p* like both the *H*^1^-broken-norm and the *L*^2^-norm errors. The numerical results confirm the theoretical estimates of Section 4 also in this 3D test case.

## Footnotes

1 It holds that $(S_{k})_{ii}={\int \limits }_{{{{\varGamma }}}_{k}}\varphi _{i}(x) dx={\sum }_{j} (M_{{{{\varGamma }}}_{k}})_{ij}$

2 We recall that $f(h)=\mathcal {O}(h^{q})$ when *h* → 0, if there exists a positive constant *c* independent of *h* such that |*f*(*h*)|≤ *c**h*^*q*^ when *h* → 0.

3 From now on, let *C* denote a constant with the aforementioned properties, but it may be different from time to time.

4 Gordon and Hall transfinite mappings [20] are used to map the reference element to the physical ones.
